# CD147 supports paclitaxel resistance via interacting with RanBP1

**DOI:** 10.1038/s41388-021-02143-3

**Published:** 2022-01-01

**Authors:** Gang Nan, Shu-Hua Zhao, Ting Wang, Dong Chao, Ruo-Fei Tian, Wen-Jing Wang, Xin Fu, Peng Lin, Ting Guo, Bin Wang, Xiu-Xuan Sun, Xi Chen, Zhi-Nan Chen, Shi-Jie Wang, Hong-Yong Cui

**Affiliations:** 1 National Translational Science Center for Molecular Medicine & Department of Cell Biology, Fourth Military Medical University, 710032 Xi’an, China; 2 Department of Obstetrics and Gynecology, Xijing Hospital, Fourth Military Medical University, 710032 Xi’an, China; 3 Department of Biochemistry and Molecular Biology, Fourth Military Medical University, 710032 Xi’an, China; 4 Department of Thoracic Surgery, the 940th hospital of joint logistics support force of Chinese People’s Liberation Army, 730050 Lanzhou, China; 5 College of Chemistry and Materials Science, Northwest University, 710127 Xi’an, China

**Keywords:** Cancer therapeutic resistance, Oncogenes, Chemotherapy, Non-small-cell lung cancer

## Abstract

Though the great success of paclitaxel, the variable response of patients to the drug limits its clinical utility and the precise mechanisms underlying the variable response to paclitaxel remain largely unknown. This study aims to verify the role and the underlying mechanisms of CD147 in paclitaxel resistance. Immunostaining was used to analyze human non-small-cell lung cancer (NSCLC) and ovarian cancer tissues. RNA-sequencing was used to identify downstream effectors. Annexin V-FITC/propidium iodide and terminal deoxynucleotidyl transferase dUTP nick end labeling (TUNEL) staining were used to detect apoptosis. Co-immunoprecipitation (Co-IP), fluorescence resonance energy transfer (FRET) and surface plasmon resonance (SPR) were performed to determine protein interactions. Fluorescence recovery after photobleaching (FRAP) was performed to measure the speed of microtubule turnover. Xenograft tumor model was established to evaluate sensitivity of cancer cells to paclitaxel in vivo. In vitro and in vivo assays showed that silencing CD147 sensitized the cancer cells to paclitaxel treatment. CD147 protected cancer cells from paclitaxel-induced caspase-3 mediated apoptosis regardless of p53 status. Truncation analysis showed that the intracellular domain of CD147 (CD147^ICD^) was indispensable for CD147-regulated sensitivity to paclitaxel. Via screening the interacting proteins of CD147^ICD^, Ran binding protein 1 (RanBP1) was identified to interact with CD147^ICD^ via its C-terminal tail. Furthermore, we showed that RanBP1 mediated CD147-regulated microtubule stability and dynamics as well as response to paclitaxel treatment. These results demonstrated that CD147 regulated paclitaxel response by interacting with the C-terminal tail of RanBP1 and targeting CD147 may be a promising strategy for preventing paclitaxel resistant.

## Introduction

Microtubules, highly dynamic components of the cytoskeleton, participate in multiple cellular activities. Assembly of mitotic microtubules into a bipolar spindle is essential for balanced chromosome distribution to newborn daughter cells at mitosis. Dysregulation of microtubule dynamics contributes to the development of serious diseases, including cancer. Microtubule dynamics is one of the major targets for chemotherapeutic agents. Paclitaxel (Taxol^®^), a member of the taxane class, is one of the most widely used chemotherapeutic agents, together with doxorubicin and cisplatin, and is first- or second-line treatment for several types of cancer, including ovarian cancer, NSCLC, breast cancer and cervical cancer. Since its approval by the FDA in 1992 for the treatment of ovarian cancer, the use of paclitaxel has led to dramatically improvement in the duration and quality of life for many cancer patients, however, the majority eventually develop progressive disease after initially responding to paclitaxel treatment. Primary or acquired resistance, which often limits the clinical utility of paclitaxel, represents a major obstacle to improving the overall response and survival of cancer patients. While a variety of mechanisms have been linked to paclitaxel resistance, including overexpression of ATP-dependent translocase ABCB1 [1], mutations in drug binding sites on tubulin [2–7], alterations in tubulin isotype distribution [8], aberrant expression of noncoding RNAs [9], impairment of apoptotic pathways [10] and altered expression of microtubule regulatory proteins [11], the mechanisms responsible for paclitaxel resistance are far from clear.

CD147, a type I transmembrane protein, belongs to the immunoglobulin superfamily. CD147, also known as extracellular matrix metalloproteinase inducer, is a multifunctional cell adhesion molecule that plays important roles in both physiological and pathological conditions, including reproduction, development, immunological responses, infectious diseases and malignant tumors [12]. CD147 is overexpressed in multiple cancer tissues, including ovarian cancer, NSCLC, breast cancer, cervical cancer and hepatocellular carcinoma [13]. Mounting evidence indicates that CD147 regulates cancer cell migration [14], invasion [15], adhesion [16], metastasis [17], angiogenesis [18], apoptosis [19, 20], autophagy [21] and proliferation [22]. Furthermore, studies showed that CD147 was associated with poor response to paclitaxel [23, 24] and inhibition of CD147 by siRNA increased chemosensitivity to paclitaxel [23], however, the mechanisms underlying CD147-mediated resistance to paclitaxel-based treatment are largely unknown.

In this study, we validated the association between CD147 expression and paclitaxel resistance in ovarian cancer and NSCLC. We demonstrated that CD147 protects cancer cells from paclitaxel-induced caspase-3-mediated apoptosis regardless of p53 status. Mechanically, we characterized RanBP1 as a binding partner for CD147^ICD^. Via binding to the C-terminal tail of RanBP1, CD147 decreased Ran-GTP and regulated microtubule stability and dynamics, which contributed to the resistance of paclitaxel treatment. These findings thus reveal CD147 as a critical regulator of paclitaxel sensitivity and have important implications in cancer chemotherapy.

## Results

### High expression of CD147 correlates with paclitaxel resistance

**Fig. 1:**
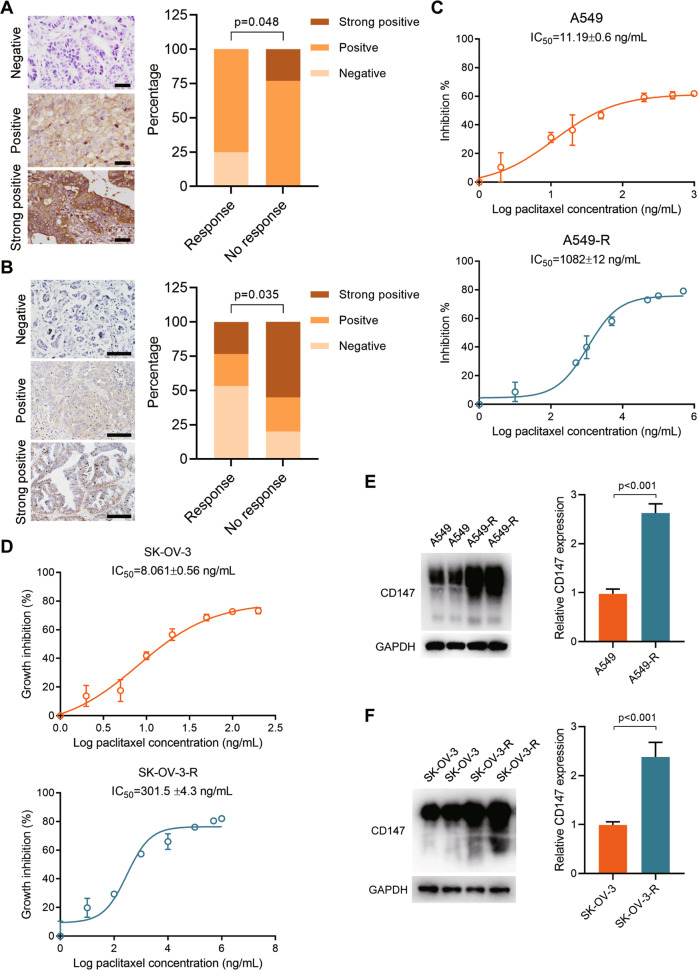
High expression of CD147 correlates with paclitaxel resistance. **A**–**B** Representative images of immunohistochemical staining of CD147 in NSCLC (**A**) and ovarian cancer (**B**) tissues. Scale bar = 200 μm. The *p* values were determined by Pearson’s chi-squared test. **C**–**D** Cytotoxic effects of increasing concentrations of paclitaxel in A549, A549-R (**C**), SK-OV-3 and SK-OV-3-R (**D**) cells. The half maximal inhibitory concentration (IC_50_) was obtained from three independent CCK-8 assays. **E**–**F** Western blot analysis of CD147 expression in the indicated cells. The graphs show semi-quantitative analysis of relative CD147 expression. The *p* values were determined by using two-tailed Student’s *t* test.

To investigate whether the expression status of CD147 is related to paclitaxel response, we determined CD147 expression using IHC in 25 NSCLC tissues (13 with no response to paclitaxel-based treatment and 12 that responded to paclitaxel-based treatment) and 50 ovarian cancer tissues (20 with no response to paclitaxel-based treatment and 30 that responded to paclitaxel-based treatment). As shown in Fig. 1A, B and Supplementary Tables 1–2, we found that the expression of CD147 was significantly higher in the paclitaxel resistant group compared to the paclitaxel sensitive group. We also generated paclitaxel resistant A549 cells (A549-R, Fig. 1C) and SK-OV-3 cells (SK-OV-3-R, Fig. 1D) and determined the expression of CD147. We found that CD147 was increased in A549-R and SK-OV-3-R cells compared to the parent cells (Fig. 1E, F). All these results indicate that CD147 may contribute to paclitaxel resistance.

### CD147 impairs induction of apoptosis by paclitaxel

**Fig. 2:**
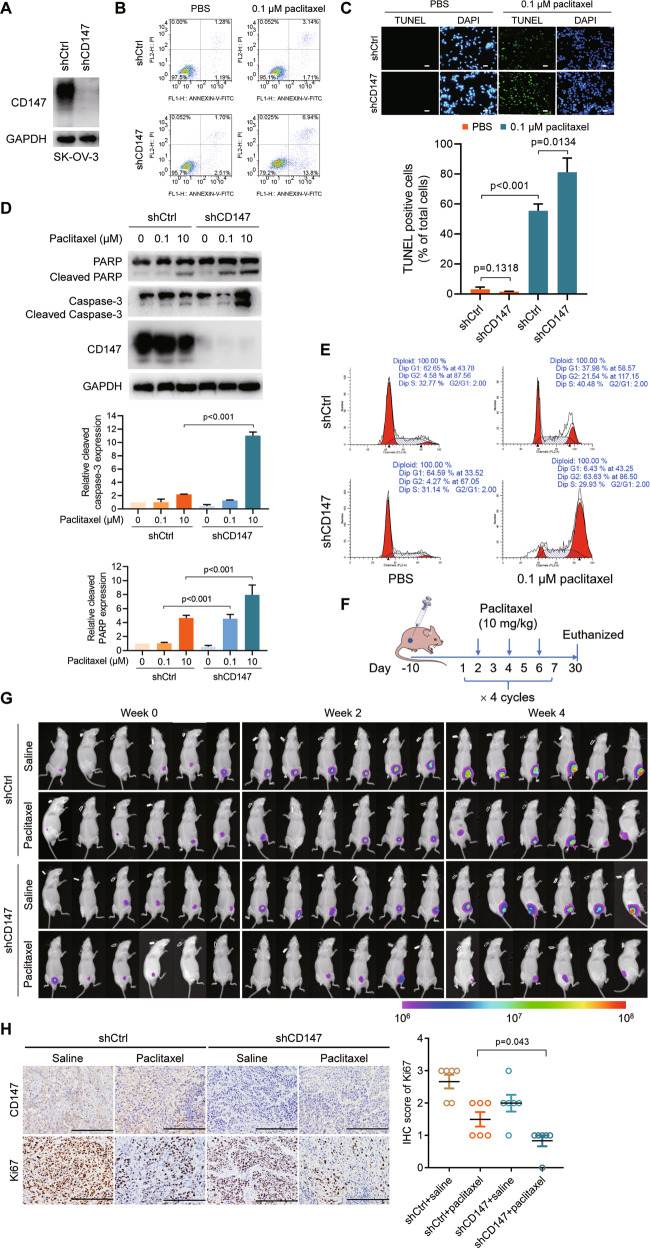
Silence of CD147 potentiates paclitaxel-induced cytotoxicity. **A** Western blot analysis of CD147 expression in SK-OV-3 cells. **B** Apoptosis analysis of SK-OV-3 cells by flow cytometry. **C** Representative images of TUNEL staining in SK-OV-3 cells. Scale bar = 20 μm. The graph shows quantification of the percentage of TUNEL positive cells. **D** Western blot analysis of the indicated proteins in SK-OV-3 cells. The graphs show semi-quantitative analysis of relative cleaved PARP and cleaved caspase-3 expression. The *p* values in (**C** and **D**) were determined by using two-tailed Student’s *t* test. **E** Cell cycle distribution of SK-OV-3 cells. **F** Schematic representation of subcutaneous cancer xenografts, as well as the schedule of paclitaxel administration in the nude mouse model. *n* = 6 for each group. **G** Nude mice with subcutaneous SK-OV-3 xenografts were imaged in a Xenogen IVIS 200 system. **H** Representative images of immunochemistry staining of CD147 and Ki67 on subcutaneous SK-OV-3 xenografts. Scale bar = 200 μm. The graph summarizes the IHC score of Ki67 and the *p* value was determined by using Mann–Whitney U test.

To further validate the role of CD147 in paclitaxel response, we generated SK-OV-3 and A549 CD147 knockdown cells (Fig. 2A and Supplementary Fig. 1A) and challenged the cells with paclitaxel. We found that paclitaxel treatment induced apoptosis (Fig. 2B–D and Supplementary Fig. 1B–D), which was consistent with the previous studies [25, 26]. Compared to the parent cells, the percentage of apoptotic cells was much higher in the CD147 knockdown cells (Fig. 2B–D and Supplementary Fig. 1B–D), which was observed in both the tested cell lines. As paclitaxel can induce mitotic arrest, we also checked the cell cycle progression. We found that paclitaxel induced G2/M phase arrest and this effect was more significant in CD147 knockdown cells (Fig. 2E and Supplementary Fig. 1E). In addition, we found that cells overexpressing CD147 were less sensitive to paclitaxel treatment (Supplementary Fig. 2). Furthermore, we performed a xenograft assay in immunodeficient mice with cells from the ovarian cancer cell line SK-OV-3 or NSCLC cell line A549 expressing scramble shRNA or shRNAs targeting CD147 (shCD147) and treated the mice with saline or paclitaxel (Fig. 2F). We found that paclitaxel treatment could decrease tumor growth. Notably, although CD147 knockdown per se delayed tumor progression to a lesser extent, the combination of CD147 knockdown and paclitaxel treatment significantly shrunk the tumors (Fig. 2G and Supplementary Fig. 1F–H), suggesting that CD147 knockdown sensitizes cancer cells to paclitaxel. Immunohistochemical staining showed that shCD147 plus paclitaxel group had weaker Ki67 staining compared to the scramble shRNA plus paclitaxel group (Fig. 2H). All these results demonstrated that CD147 decreases sensitivity of cancer cells to paclitaxel.

### Caspase-3 regulates apoptosis induction by paclitaxel in CD147-silenced cells

**Fig. 3:**
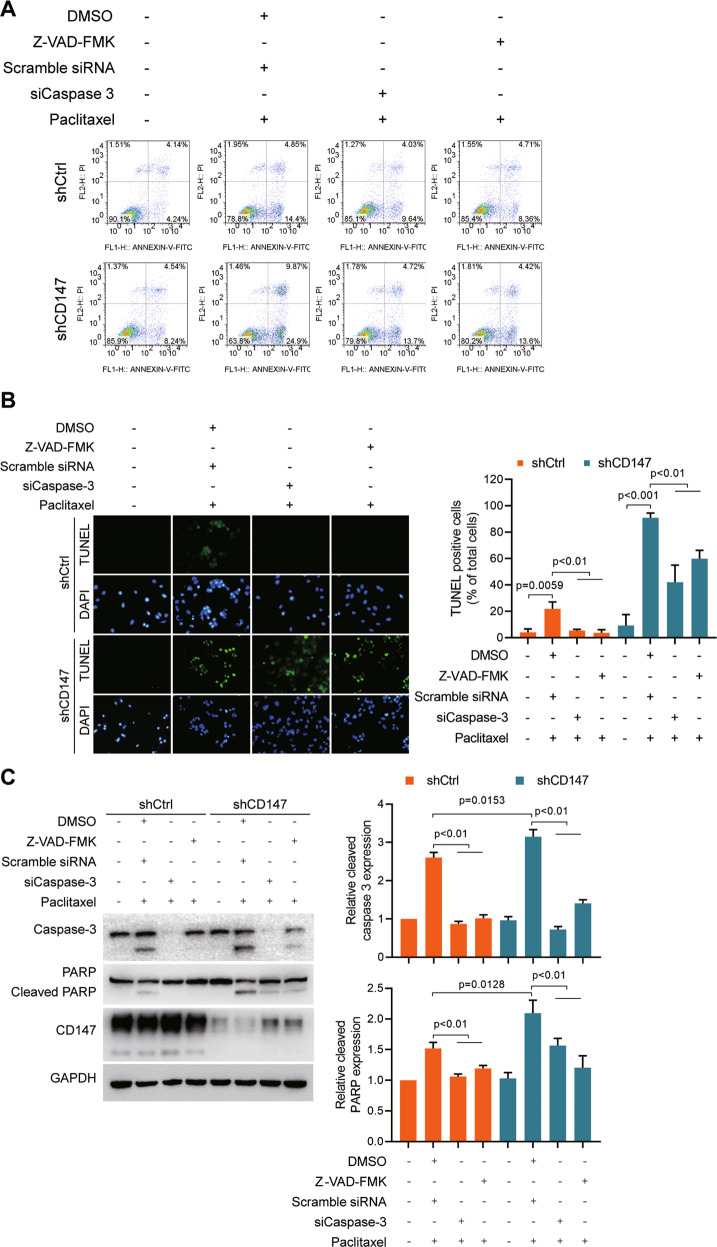
Caspase-3 regulates apoptosis induction by paclitaxel in CD147-silenced cells. **A** Analysis of apoptosis by flow cytometry. SK-OV-3 cells with stably silencing of CD147 (shCD147) or control cells (shCtrl) were transfected with siRNAs targeting caspase 3 (siCaspase 3) or scramble siRNA alone or in combination with 40 μM Z-VAD-FAK treatment. **B** Representative images of TUNEL staining of SK-OV-3 cells. Scale bar = 20 μm. The graph shows quantification of the percentage of TUNEL positive cells. **C** Western blot analysis of the indicated proteins in SK-OV-3 cells. The graphs show semi-quantitative analysis of relative cleaved PARP and cleaved caspase-3 expression. The *p* values in (**B** and **C**) were determined by using two-tailed Student’s *t* test.

Because caspase-3 enzyme is a member of the family of endoproteases which regulate apoptosis signaling network. We determined whether caspase-3 regulated paclitaxel-induced apoptosis in CD147 knockdown cells. We found that siRNAs targeting caspase-3 as well as pan caspase inhibitor Z-VAD-FMK could relieve paclitaxel-induced apoptosis in CD147 knockdown cells (Fig. 3 and Supplementary 3). Notably, we found that CD147 knockdown sensitized cancer cells to paclitaxel and Z-VAD-FMK could relieve paclitaxel-induced apoptosis in A549 cells treated with p53 inhibitor Pifithrin-α as well as SK-OV-3 cells overexpressing p53 (Supplementary Fig. 4). All these results suggest that CD147 protects cancer cells from paclitaxel-induced caspase-3 mediated apoptosis regardless of p53 status.

### CD147 regulates microtubule dynamics

**Fig. 4:**
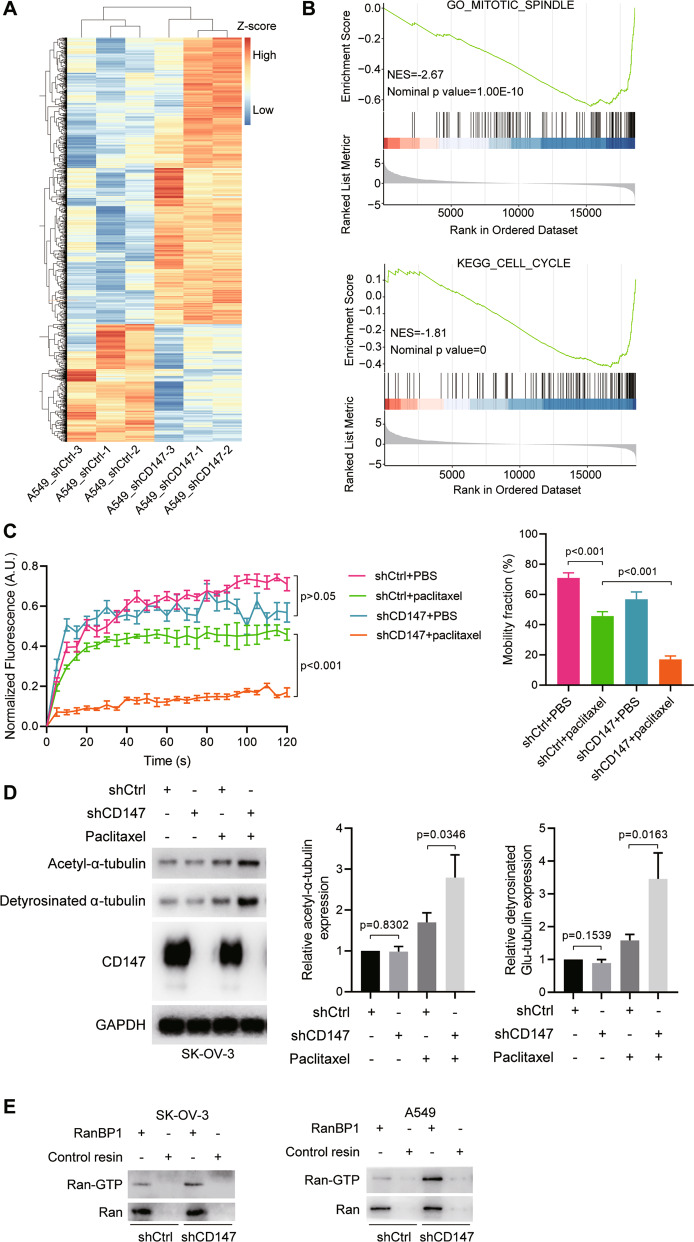
CD147 knockdown promotes paclitaxel-induced stabilization of microtubules. **A** Heatmap of DEGs in CD147 knockdown A549 cells (A549_shCD147) and control cells (A549_shCtrl). Cells were treated with 0.1 μM paclitaxel for 48 h. **B** Gene set enrichment analysis of DEGs. **C** Analysis of FRAP assay. The *p* values were determined by one-way ANOVA with Tukey’s post hoc test. **D** Western blot analysis of the indicated proteins in CD147 knockdown cells and control cells treated with or without 0.1 μM paclitaxel. The graphs show semi-quantitative analysis of relative acetyl-α-tubulin and detyrosinated α-tubulin expression. The *p* values were determined by using two-tailed Student’s *t* test. **E** Western blot analysis of Ran-GTP in CD147 knockdown cells and control cells.

To explore the mechanisms by which CD147 regulates the sensitivity of cancer cells to paclitaxel, we employed gene expression profiling in cancer cells expressing scramble shRNA or shRNAs targeting CD147, which were challenged with paclitaxel. In the NSCLC cell line A549, of the 2059 differentially expressed genes (DEGs) identified, 1103 genes were upregulated and 956 genes were down-regulated (Fig. 4A and Supplementary Fig. 5A). Gene set enrichment analysis showed that the gene set apoptosis was upregulated in A549_shCD147 cells (Supplementary Fig. 5B) and the gene sets mitotic spindle and cell cycle were upregulated in A549_shCtrl cells (Fig. 4B). In ovarian cancer cell line SK-OV-3, of the 6072 DEGs identified, 3403 genes were upregulated and 2669 genes were down-regulated (Supplementary Fig. 5C, D). Gene set enrichment analysis showed that the gene set mitotic cell cycle arrest was upregulated in SK-OV-3_shCD147 cells and the gene set mitotic spindle was upregulated in SK-OV-3_shCtrl cells (Supplementary Fig. 5E). All these data indicate that CD147 may alleviate the cytotoxicity of paclitaxel by regulating spindle formation. Given paclitaxel exerts its action by directly inducing the formation of static microtubules and spindle formation is driven by dynamic microtubules and RanGTP gradient, we checked the effects of CD147 knockdown on microtubule dynamics and RanGTP. We applied live-cell microscopy to record microtubule dynamics in A549 cells stably transfected GFP-α-tubulin. We used the FRAP technique to measure the speed of microtubule turnover. As expected, cells responded to paclitaxel treatment with a reduced mobile fraction after photobleaching. In contrast, cells with CD147 knockdown were more responsive to paclitaxel (Fig. 4C). In the second approach, we analyzed two forms of posttranslational modifications of α-tubulin, acetylated α-tubulin at Lys40 and detyrosinated α-tubulin, which were less dynamic and more stable. Consistent with the pharmacological activity of paclitaxel, we found that paclitaxel treatment resulted in increased acetylated α-tubulin and detyrosinated α-tubulin, indicating increased microtubule stability. Notably, we found that acetylated α-tubulin and detyrosinated α-tubulin induced by paclitaxel treatment were further increased in CD147 knockdown cells (Fig. 4D and Supplementary Fig. 5F). Thus, these results confirmed that CD147 knockdown promoted paclitaxel-induced stabilization of microtubules. In addition, we examined the Ran-GTP level and found that CD147 knockdown cells had higher Ran-GTP level (Fig. 4E). All these results suggest that silence of CD147 in the presence of paclitaxel augments microtubule stabilization and cytotoxicity.

### The intracellular domain of CD147 (CD147^ICD^) is indispensable for CD147-regulated paclitaxel resistance

**Fig. 5:**
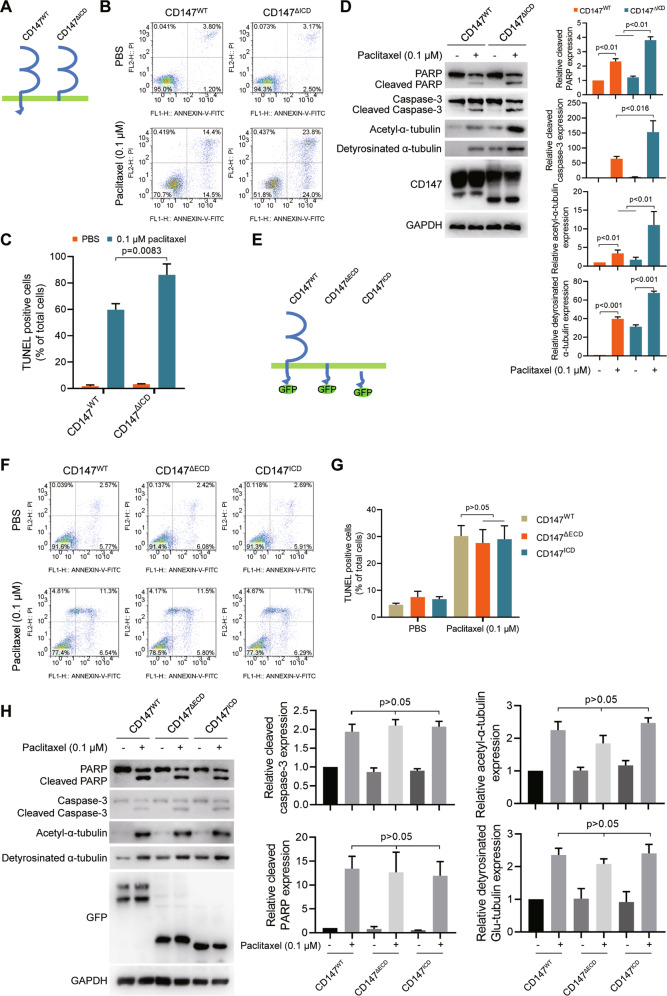
The intracellular domain of CD147 is indispensable for CD147-regulated paclitaxel resistance. **A** Schematic representation of the CD147 constructs. **B** Analysis of apoptosis by flow cytometry. CD147 knockdown SK-OV-3 cells were transfected with CD147^WT^ or CD147^ΔICD^. Cells were treated with PBS or 0.1 μM paclitaxel. **C** The graph shows quantification of the percentage of TUNEL positive cells. **D** Western blot analysis of the indicated proteins in CD147 knockdown SK-OV-3 cells. Cells were transfected with CD147^WT^ or CD147^ΔICD^ and treated with or without 0.1 μM paclitaxel. The graphs show semi-quantitative analysis of protein expression. **E** Schematic representation of the CD147 constructs. **F** Analysis of apoptosis by flow cytometry. CD147 knockdown SK-OV-3 cells were transfected with CD147^WT^, CD147^ΔECD^ or CD147^ICD^. Cells were treated with PBS or 0.1 μM paclitaxel. **G** The graph shows quantification of the percentage of TUNEL positive cells. **H** Western blot analysis of the indicated proteins in CD147 knockdown SK-OV-3 cells. Cells were transfected with CD147^WT^, CD147^ΔECD^ or CD147^ICD^ and treated with or without 0.1 μM paclitaxel. The graphs show semi-quantitative analysis of protein expression. The *p* values in (**C**–**D**), (**G**–**H**) were determined by two-tailed Student’s *t* test.

To determine which domain of CD147 is critical for regulating paclitaxel sensibility, we restored the expression of wild-type CD147 (CD147^WT^) or truncated CD147 lacking the intracellular domain (CD147^ΔICD^) in CD147 knockdown cells (Fig. 5A) and found that the cells expressing CD147^ΔICD^ were more sensitive to paclitaxel treatment compared with the cells expressing CD147^WT^ (Fig. 5B–D and Supplementary Fig. 6A–C). Furthermore, we restored the expression of wild-type CD147 (CD147^WT^), truncated CD147 lacking the extracellular domain (CD147^ΔECD^) or the intracellular domain of CD147 (CD147^ICD^) in CD147 knockdown cells (Fig. 5E) and found that the cells expressing CD147^ΔECD^ or CD147^ICD^ were less sensitive to paclitaxel treatment compared with the cells expressing CD147^WT^ (Fig. 5F–H and Supplementary Fig. 6D, E). These results indicate that the intracellular domain of CD147 is responsible for CD147-regulated paclitaxel response.

### CD147^ICD^ interacts with RanBP1

**Fig. 6:**
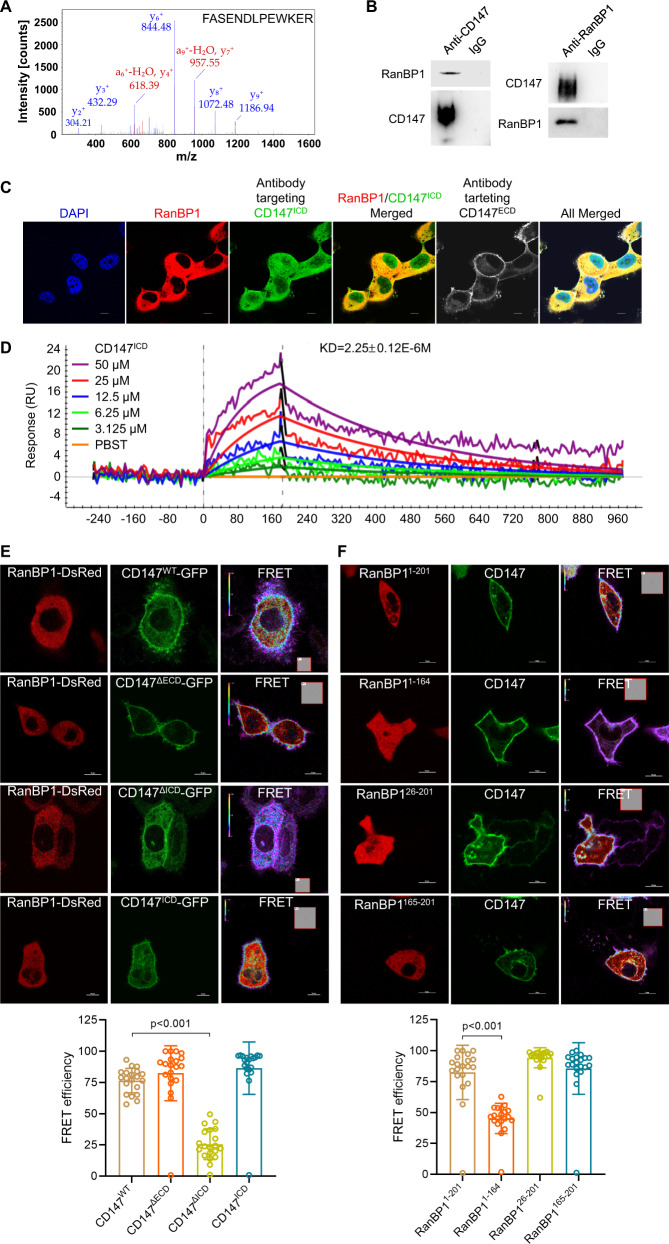
CD147^ICD^ interacts with RanBP1. **A** MS/MS confirmation of a RanBP1 peptide. **B** Western blot analyses of endogenous CD147 co-immunoprecipitated with endogenous RanBP1 in SK-VO-3 cells. IgG was used as a control antibody for immunoprecipitation. **C** Representative images of immunofluorescent staining of endogenous CD147 and RanBP1 in A549 cells. Scale bar = 10 μm. **D** SPR diagram of RanBP1 protein bound to CD147^ICD^. The KD values were calculated by the the ProteOn Manager Software. **E**–**F** The interaction between the indicated constructs was analyzed with FRET. The color bar represents the FRET ratio. Scale bar, 10 μm. The graphs show FRET efficiencies. The *p* values were determined by two-tailed Student’s *t* test.

We showed that CD147 regulates paclitaxel resistance via its intracellular domain. We next tried to identify the CD147^ICD^-associated molecules responsible for this regulation. A His pull-down assay was employed to capture the proteins interacting with CD147^ICD^, where purified His_6_-tagged CD147^ICD^ was the bait (Supplementary Fig. 7A), and the lysates from A549 or SK-VO-3 cells were prey (Supplementary Fig. 7B). Through LC-MS/MS analysis, overlapping proteins interacting with the control resin were removed, and RanBP1 emerged as having the highest probability of being a candidate binding partner for CD147^ICD^ (Fig. 6A, Supplementary Table 3). We showed that endogenous CD147 could form a complex with RanBP1 in SK-VO-3 cells (a reciprocal Co-IP assay, Fig. 6B) and A549 cells (Supplementary Fig. 7C). The co-localization of CD147 and RanBP1 in A549 cells (Fig. 6C) and SK-OV-3 cells (Supplementary Fig. 7D) as well as cancer tissues was confirmed via immunofluorescence staining (NSCLC tissue, Supplementary Fig. 7E; ovarian cancer tissue, Supplementary Fig. 7F). Notably, the co-localization of RanBP1 and CD147 was more significant when using a monoclonal antibody targeting the intracellular domain of CD147 (anti-CD147^ICD^) rather than the monoclonal antibody targeting the extracellular domain of CD147 (anti-CD147^ECD^). In addition, we showed that CD147^ICD^ directly interacted with RanBP1 by using SPR assay with purified proteins (KD = 2.25 ± 0.12 μM, Fig. 6D). Furthermore, FRET assays showed that CD147^WT^, CD147^ΔECD^ and CD147^ICD^ but not CD147^ΔICD^ could interact with RanBP1 in live cells (Fig. 6E). We also truncated RanBP1 (Supplementary Fig. 7G) and FRET assays showed that the C-terminal tail (amino acids 165–201) of RanBP1 mediates binding of RanBP1 to CD147 (Fig. 6F). Co-IP assays confirmed the interaction between the C-terminal tail of RanBP1 and CD147 in A549 cells (Supplementary Fig. 7H). All these results suggest that the intracellular domain of CD147 interacts with the C-terminal tail of RanBP1.

### CD147 regulates microtubule stability via RanBP1

**Fig. 7:**
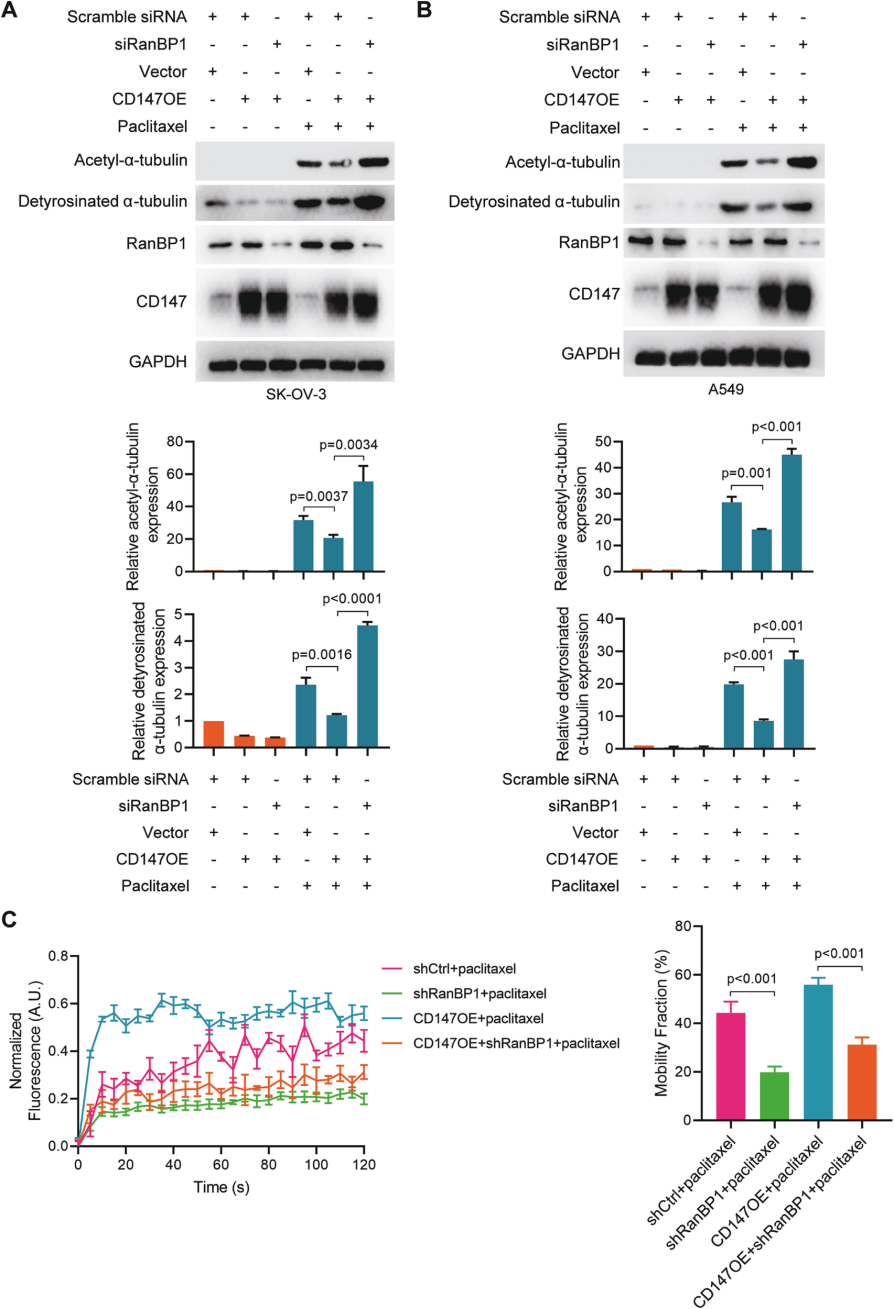
CD147 regulates tubulin stability via RanBP1. **A**–**B** Western blot analysis of the indicated proteins in SK-OV-3 (**A**) and A549 (**B**) cells treated with or without 0.1 μM paclitaxel. Cell were transfected with CD147-pcDNA3.1 or vector alone, or in combination with either siRNA targeting RanBP1 (siRanBP1) or scramble siRNA. The graphs show semi-quantitative analysis of relative protein expression. The *p* values were determined by using two-tailed Student’s *t* test. **C** FRAP assay analysis of mobility fraction. The *p* values were determined by one-way ANOVA with Tukey’s post hoc test.

Since the Ran GTPase plays critical roles in regulating microtubule cytoskeleton dynamics and RanBP1 has been characterized as a coactivator of the Ran GTPase-activating protein (RanGAP1) and regulates Ran-GTP production. To investigate whether CD147 regulates microtubule stability and dynamics via RanBP1, we overexpressed CD147 and silenced RanBP1. As shown in Fig. 7, we found that CD147 overexpression impaired the effects of paclitaxel treatment on acetylated α-tubulin and detyrosinated α-tubulin as well as patterns of microtubule dynamics. Notably, the impairment induced by CD147 overexpression was restored, at least partially, by RanBP1 silence. All these results indicate that CD147 is involved in regulation of microtubule stability and dynamics and this regulation is mediated by RanBP1.

### CD147 regulates paclitaxel response via RanBP1

**Fig. 8:**
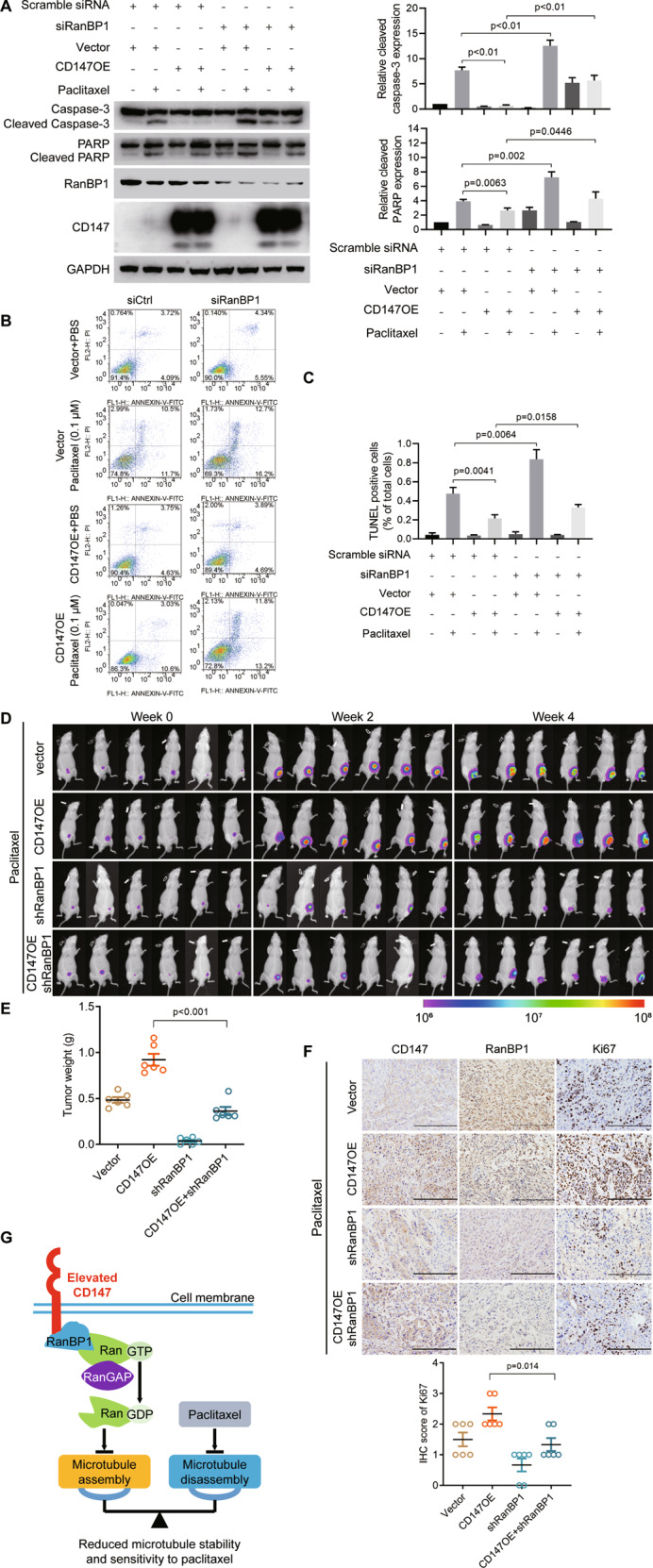
CD147 decreases paclitaxel-induced cytotoxicity via RanBP1. **A** Western blot analysis of the indicated proteins in SK-OV-3 cells treated with or without 0.1 μM paclitaxel. Cells were transfected with CD147-pcDNA3.1 or in combination with either siRNA targeting RanBP1 (siRanBP1) or scramble siRNA. The graphs show semi-quantitative analysis of relative cleaved PARP and cleaved caspase-3 expression. The *p* values were determined by using two-tailed Student’s *t* test. **B** Analysis of apoptosis by flow cytometry. Cells were transfected with CD147-pcDNA3.1 or in combination with either siRNA targeting RanBP1 (siRanBP1) or scramble siRNA. **C** The graph shows quantification of the percentage of TUNEL positive cells. **D** Nude mice with subcutaneous ovarian cancer xenografts were imaged in a Xenogen IVIS 200 system. **E** Quantification of tumor weight after subcutaneous implantation of ovarian cancer cells. **F** Representative images of immunochemistry staining of CD147, RanBP1 and Ki67 in subcutaneous tumors of nude mice. Scale bar = 200 μm. The graph summarizes the IHC score of Ki67. The *p* values in (**A**, **C**, **E** and **F**) were determined by two-tailed Student’s *t* test. **G** Model depicting the proposed mechanism mediating paclitaxel response.

To determine whether CD147 regulates paclitaxel response via RanBP1, we treated cancer cells stably overexpressing CD147 with paclitaxel and silenced RanBP1 by siRNAs. As shown in Fig. 8A–C and Supplementary Fig. 8A–C, we found that CD147 overexpression enhanced cell viability and decreased apoptosis when treated with paclitaxel. Contrary to CD147 overexpression, silence of RanBP1 led to decreased cell viability and increased apoptosis. Notably, silence of RanBP1 abolished the effects of CD147 overexpression on cell viability and apoptosis. All these results indicate that RanBP1 mediates the effects of CD147 on paclitaxel response.

### CD147 regulates paclitaxel response via RanBP1 in vivo

To further investigate the in vivo role of RanBP1 in CD147-modulated paclitaxel response, we performed a xenograft assay in immunodeficient mice with cells from the ovarian cancer cell line SK-OV-3 overexpressing CD147 and/or shRNAs targeting RanBP1 (shRanBP1). When treated with paclitaxel, we found that CD147 overexpression group was less sensitive to paclitaxel compared with the control group. Notably, silence of RanBP1 significantly sensitized the cells to paclitaxel and reversed the effects of CD147 overexpression on paclitaxel response (Fig. 8D–E and Supplementary Fig. 8D). Using cells from the NSCLC cell line A549 overexpressing CD147 and/or shRanBP1 we repeated the experiment and obtained similar results (Supplementary Fig. 9). Further analysis showed that CD147 overexpression group had more Ki67 expression (Fig. 8F), indicating that the cells overexpressing CD147 were more resistant to paclitaxel. On the contrary, shRanBP1 group showed minimal Ki67 expression. All these data suggest that CD147-modulated paclitaxel response is mediated by RanBP1.

## Discussion

CD147 is a multifunctional oncogenic protein. We and others have shown that CD147 is involved in chemotherapy response and resistance. CD147 silencing increased the sensitivity of cancer cells to 5-fluorouracil in breast cancer [27] and oral squamous carcinoma cells [20]. CD147 inhibited hepatocellular carcinoma cell apoptosis and decreased adriamycin chemosensitivity by inducing the unfolded protein response [19]. Anti-CD147 antibody sensitized pancreatic cancer cells to gemcitabine and genfitinib by blocking CD44s-signal transducer and activator of transcription 3 signaling [28]. CD147 was identified as a strong independent prognostic factor for response and survival after cisplatin-containing chemotherapy in patients with advanced bladder cancer [29]. The expression of CD147 and Lewis y antigen was an independent risk factor for paclitaxel and carboplatin resistance in ovarian cancer [30]. In the present study, we provide several lines of evidence demonstrating the involvement of CD147 in regulating cancer cell sensitivity to paclitaxel: (a) CD147 expression in ovarian cancer and NSCLC tissues correlates with pathological response of tumors to paclitaxel-based treatment; (b) CD147 expression increases in paclitaxel resistant cells compared with the parent cells, though the mechanism underlying CD147 upregulation remains to be elucidated; and (c) depletion of CD147 increases paclitaxel sensitivity and overexpression of CD147 has the opposite effect. Our results suggest a possibility of improving the pathological response to paclitaxel through modulating CD147 expression.

Paclitaxel is a potent mitotic poison, which binds to β-tubulin in the α-β-tubulin heterodimer and suppresses spindle microtubule dynamics, causing a delay or block at the metaphase-anaphase transition during mitosis. Disruption of the mitotic spindle dose not satisfy the spindle assembly checkpoint, which causes an extended mitotic arrest that can lead to cell death [31]. Given the preliminary pharmacological mechanism of paclitaxel, it is not surprising to speculate that proteins with regulatory effects on microtubule dynamics and/or spindle assembly may antagonize or synergize the anti-tumor activity of paclitaxel, and the dysregulation of these proteins may contribute to the variable response to paclitaxel treatment. Actually, previous work has shown that microtubule dynamics of cancer cells have a profound effect on the magnitude of paclitaxel response. For example, inactivation of tyrosine-protein kinase Fer increases microtubule stability in ovarian cancer cells, potentiating the cytotoxicity of paclitaxel [32]. Downregulation of PI3K-C2α causes spindle alterations and increases sensitivity to taxane-based therapy [33]. Here, we provide another example, CD147 decreases microtubule stability and supports resistance to paclitaxel treatment (Fig. 8G).

We have also explored the potential mechanism of how CD147 regulates paclitaxel sensitivity. CD147 is a type I transmembrane protein and previous studies mainly focus on the extracellular portion of CD147, where CD147 serves as a receptor for several molecules through trans-recognition using its extracellular Ig domains and recognizes molecules in the same cells [12]. Here, we demonstrated that CD147^ICD^ but not CD147^ECD^ is responsible for regulating sensibility to paclitaxel. Through screening the interacting partners of CD147^ICD^ and functional verification, RanBP1 was characterized as a direct interacting partner for CD147^ICD^ and mediated CD147-induced resistance to paclitaxel. Recently, we found that CD147^ICD^ can be released by two ways: intramembranous cleavage by the γ-secretase at lysine 231 [34] and procession of residual CD147 in the lysosome after shedding of CD147^ECD^ [35]. Thus, the released CD147^ICD^ may bind to RanBP1 after mitotic nuclear membrane breakdown and regulate sensitivity to paclitaxel. As a specific antibody targeting CD147^ECD^ was found to inhibit CD147 shedding and sequential CD147^ICD^ release, this might be a part of the reason why CD147 antibody could enhance the sensitivity of cancer cells to paclitaxel [36].

Ras-related nuclear protein (Ran) is a small GTPase that controls multiple cellular processes, including mitotic spindle assembly [37]. The Ran gradient is highly regulated during mitosis. After mitotic nuclear membrane breakdown, regulator of chromosome condensation (RCC1) generates Ran-GTP near mitotic chromosomes, while the majority of Ran distal to chromosomes is GDP-bound [38]. The presence of a mitotic Ran-GTP gradient directs proper spindle assembly by releasing spindle assembly factors in a spatially regulated manner [39]. RanBP1, a Ran-GTP binding protein found in many eukaryotes, stimulates RanGAP1’s activity roughly tenfold within in vitro assays using purified proteins [40]. It also promotes Ran-GTP release from karyopherin, thereby further enhancing RanGAP1-activated GTP hydrolysis on Ran [41, 42]. Structure analysis of the Ran-RanBP1-RanGAP ternary complex indicates that RanBP1 does not directly modify the properties of RanGAP and it functions by relieving the inhibition of RanGAP by the C terminus of Ran [43]. RanBP1 can form a stable heterotrimeric complex with Ran and RCC1 in vitro, inhibiting RCC1’s nucleotide exchange activity [40]. Recent studies indicate that RanBP1 promotes accurate chromosome segregation through maintenance of optimal Ran-GTP gradients during mitosis in mammalian cells [44]. In the present study, we demonstrated that CD147^ICD^ directly interacted with the C-terminal tail of RanBP1 and overexpression of CD147 leads to the decreased abundance of Ran-GTP. One possible explanation could be that CD147 binds to the C-terminal tail of RanBP1 and induces conformation change of RanBP1, resulting in increased association between Ran and RanBP1 and promoting disinhibition of RanGAP. However, more conclusive evidence of RanBP1 conformation change induced by CD147 binding depends on structural studies.

In conclusion, we revealed that enhanced expression of CD147 regulated microtubule stability and dynamics via interacting with the C-terminal tail of RanBP1, which contributed to reduced sensitivity to paclitaxel treatment. Moreover, the in vitro and in vivo results may provide a rational for decreasing CD147 as a new therapeutic strategy to enhance sensitivity to paclitaxel-containing chemotherapy.

## Materials and methods

### Cell lines

The A549 and SK-OV-3 cell lines were purchased from American Type Culture Collection and cultured in RPMI 1640 medium supplemented with 10% fetal bovine serum. CD147 knockdown and RanBP1 knockdown stable cell lines were generated using lentivirus and the stable cell pool was used for biological assays. The target sequences were depicted in Supplementary Table 4. CD147 overexpression cells were generated by transfecting cells with CD147/pcDNA3.1(+). Cells stably expressing EGFP-tagged α-tubulin were generated by transfecting cells with α-tubulin/pEGFP-N1. The paclitaxel resistant A549 cells (A549-R) and SK-OV-3 cells (SK-OV-3-R) were generated by challenging the cells with increasing amounts of paclitaxel. CCK-8 assays were performed to confirm resistance to paclitaxel. All cells were cultured at 37 °C and with 5% CO_2_. All cells were authenticated using Short Tandem Repeat DNA profiling by Beijing Microread Genetics (Beijing, China) and routine mycoplasma testing was performed by PCR.

### Human cancer tissues

Human ovarian cancer tissues were collected from the Department of Obstetrics and Gynecology, Xijing Hospital, which is affiliated with the Fourth Military Medical University. Human NSCLC tissues were collected from the Department of Thoracic Surgery, the 940th hospital of joint logistics support force of Chinese People’s Liberation Army. All individuals provided written informed consent, and the study was approved by the Hospital Ethics Committee.

### Immunohistochemistry (IHC)

Tissues were fixed with 10% formalin and embedded in paraffin. Sections were deparaffinized and rehydrated. Antigen demasking was performed with citrate buffer. Sections were incubated overnight with primary antibodies at the appropriate dilutions and then incubated with biotinylated secondary antibodies for 1 h at room temperature. Visualization was performed using the DAB Horseradish Peroxidase Color Development Kit (ZSGB-BIO, Beijing, China). The expression levels were independently evaluated by two senior pathologists according to the proportion and intensity of positive cells. The following criteria were used to score each specimen: 0 (no staining), 1 (any percentage with weak intensity or <30% with strong intensity), 2 (30–50% with strong intensity), and 3 (>50% with strong intensity). Scores 0 and 1 were defined as negative and positive, respectively. Scores 2 and 3 were defined as strong positive.

### Western blot

Tissues or cell pellets were lysed with ice-cold RIPA buffer (Beyotime, Shanghai, China) containing protease and phosphatase inhibitors (Roche, Basel, Switzerland). Equal amounts of protein were separated by 10% or 12% SDS-PAGE, and transferred to PVDF membranes (Millipore, MA, USA). The membranes were blocked and then incubated with primary and secondary antibodies according to the manufacturer’s instructions. The immunoblots were developed using an ECL kit (Beyotime). Signal detection was conducted using a ChemiDoc^™^ Touch Imaging System and analyzed using Image Lab^™^ Software (Bio-Rad, CA, USA). The antibodies used in this study were listed in Supplementary Table 5.

### CCK-8 assay

Cells (1 × 10^3^) were seeded in 96-well plates with five duplicate wells in each group. Cells were treated with or without paclitaxel at various concentrations. Then CCK-8 solution (10 μL) at a 1:10 dilution with serum-free RPMI 1640 (100 μL) was added to each well, followed by a further 2 h incubation under 5% CO_2_ at 37 °C. The absorbance was automatically measured at 450 nm with a microplate reader (Epoch, BioTek Instruments, VT, USA). Three different experiments were performed for each experimental condition.

### Apoptosis and cell cycle assays

Apoptosis and cell cycle rates were assessed using an Annexin V-FITC/propidium iodide apoptosis detection kit and a cell cycle detection kit, respectively (KeyGEN Biotech, Nanjing, China). Quantification of propidium iodide and FITC signals was performed using a fluorescence activated cell sorter FACSAria system (BD Bioscience, NJ, USA).

### TUNEL staining

TUNEL staining was performed using the one-step TUNEL apoptosis kit (Beyotime) according to the manufacturer’s instructions. The cells with green fluorescence were defined as apoptotic cells. The percentage of TUNEL-positive cells relative to DAPI-stained cells was calculated.

### RNA sequencing

Total RNA was extracted using Trizol (TIANGEN, Shanghai, China). Sequence libraries were generated and sequenced by BGI (Shenzhen, China). The clean reads were subsequently aligned to the reference genome using HISAT2 (Johns Hopkins University, MD, USA) with default parameters. The processed reads from each sample were aligned using HISAT (Johns Hopkins University) against the corresponding human reference genome. Gene expression analyses were performed using Cuffquant and Cuffnorm (Cufflinks 2.2.1). Cuffdiff was used to analyze the DEGs between samples. The parameters for classifying significantly DEGs was ≥2-fold difference (|log2FC | ≥ 1, FC: fold change of expression) in transcript abundance. Gene set enrichment analysis was performed using Gene Set Enrichment Analysis software version 4.0.3 [45].

### Protein purification

The DNA sequence encoding CD147^ICD^ (residues 231–269) was subcloned into the pET21a vector (Merck, NJ, USA) with a SUMO-His tag at the C-terminus. *Escherichia coli* strain BL21 (DE3) harboring the construct was cultured in LB medium with ampicillin (50 μg/mL) at 37 °C until the OD600 ≈ 0.6, and protein expression was induced with 100 μM IPTG at 18 °C overnight. The cells were lysed by freeze-thaw cycles followed by sonication. After centrifugation, the supernatant was applied to HisTrap HP column (GE, MA, USA). The SUMO tag was removed by overnight digestion with Ulp1 protease. The proteins were further purified through size exclusion chromatography on a Superdex 75 column (GE) in 50 mM PBS (pH 7.5).

The DNA sequence encoding full-length RanBP1 was subcloned into the pMAL-c5x vector (NEB, MA, USA) to generate RanBP1 protein with an N-terminal soluble MBP tag that promotes recombinant protein secretion to the periplasm. The *Escherichia coli* strain BL21 (DE3) harboring the construct was cultured in LB medium at 37 °C until OD600 ≈ 0.6, and protein expression was induced with 26.64 μM arabinose for 6 h at 37 °C. The cells were harvested by centrifugation, resuspended in binding buffer (20 mM Tris-HCl, 200 mM NaCl, and 1 mM EDTA, pH 7.4), and lysed by freeze-thaw cycles followed by sonication. After centrifugation, the supernatant was applied to an MBPTrap HP column (GE). After digestion with thrombin (JingKe, Beijing, China), proteins were further purified through size exclusion chromatography on a Superdex 75 column (GE) in 50 mM PBS (pH 7.5).

### SPR

SPR studies were performed using a ProteOn XPR36 (Bio-Rad) instrument according to a One-shot Kinetics protocol. A concentration series of CD147^ICD^ was injected over the immobilized ligand. Injection of the analyte diluted in TBS-T was done in the horizontal direction at 50 μL/min for 3 min. An equivalent buffer injection was used for reference subtraction. The dissociation time was set to 12 min. Equilibrium and rate constants were calculated using ProteOn Manager Software.

### FRET assay

Three sequential images were acquired in the same field with suitable filter sets for the donor (EGFP; excitation at 488 nm and emission at 515 nm), acceptor (DsRed; excitation at 543 nm and emission at 585 nm), and FRET (excitation at 488 nm and emission at 585 nm). FRET calibration and net FRET were calculated using analysis software (Nikon, Tokyo, Japan).

### Immunofluorescence staining

Cells were harvested and allowed to attach for 24 h to cell culture dishes with glass bottoms (NEST, Wuxi, China). After being washed twice with PBS, the cells were fixed in paraformaldehyde in PBS, permeabilized with 0.1% Triton X-100, and blocked with 1% bovine serum albumin in PBS for 1 h. The cells were then incubated with the indicated primary antibodies for 1 h, washed twice with PBS, and incubated with the appropriate secondary antibodies according to the manufacturer’s instructions. Cell nuclei were stained with DAPI (Vector Labs, CA, USA). After washing, the cells were visualized using an A1R-A1 confocal laser microscope system (Nikon).

### Ran-GTP determination

Ran-GTP was determined using a Ran activation assay kit (Abcam, Cambridge, UK). The cells were washed twice with ice-cold PBS and lysed with 1× Assay Buffer. 40 μL of resuspended RanBP1 bead slurry was added to each tube of samples. The tubes were incubated at 4 °C for 1 h with gentle agitation. After washing the bead pellet three times with 1× Assay Buffer, the bead pellet was resuspended in 40 μL 2× reducing SDS-PAGE sample buffer. The pull-down supernatant was analyzed by western blot.

### Co-IP assay

Co-IP assay was performed using a Co-IP kit (ThermoFisher, MA, USA) as previously described [46]. Briefly, 15 μg of affinity-purified antibody was coupled to the resin. The antibody-agarose beads were mixed with the lysate, and the mixture was incubated with gentle mixing at 4 °C overnight. After washing with ice-cold IP lysis/wash buffer for six times, the immunoprecipitated proteins were then eluted and detected via western blot.

### FRAP

FRAP was performed as previously described [47]. Briefly, A549 cells were transfected with a plasmid expressing GFP-α-tubulin, and multiple positive clones were pooled for further expansion. Photobleaching procedures were performed using an A1R-A1 confocal laser microscope system (Nikon). Regions of interest were bleached for 10 s followed by 2 min of observation, with image acquisition every 2 s. At least 20 observations were recorded per sample, and the recovered fluorescence intensities were normalized against background and unbleached regions within the cell of interest.

### Xenograft tumor model

All animal protocols were approved by the Animal Care and Welfare Committee of the Fourth Military Medical University. The investigator was not blinded during the experiment. Cells used for in vivo assays were infected with lentivirus expressing firefly luciferase (Genechem, Shanghai, China). Mice were randomly allocated among groups. In total, 2 × 10^6^ cells in 0.1 mL RPMI 1640 medium were subcutaneously inoculated into the right posterior flank of male BALB/c nude mice (~20 g, *n* = 6 per group). Ten days later, the mice were treated with intraperitoneal injection of saline or paclitaxel (10 mg/ kg) 3 times/week for 4 weeks. At 1 h, 2 weeks and 4 weeks after treatment, the in vivo fluorescence images of the mice after they were anesthetized with isoflurane were acquired using the IVIS imaging system (PerkinElmer, MA, USA). After 4 weeks of treatment, the mice were anesthetized and euthanized by cervical dislocation, and the tumor tissues were analyzed by hematoxylin and eosin and immunohistochemical staining.

### Statistical analyses

Quantitative results are presented as the mean ± SD. The sample size was determined based on our experience and no statistical method was used to predetermine the sample size. The normality of the data distributions was checked using the Shapiro–Wilk test. Statistical analyses are described for each panel. All reported *p* values were two-tailed, and *p* < 0.05 was considered statistically significant.

## Supplementary information

Supplementary Information
