# Supplementary material for: CD147 supports paclitaxel resistance via interacting with RanBP1
Source: Oncogene. 2022 Jan 1;41(7):983–96. doi: 10.1038/s41388-021-02143-3 (PMC8837534; doi:10.1038/s41388-021-02143-3)
Supplement: Supplementary file 1 — Supplementary Information [file 41388_2021_2143_MOESM1_ESM.docx]

Supplementary Information for

CD147 supports paclitaxel resistance via interacting with RanBP1

Gang Nan, Shu-Hua Zhao, Ting Wang, Dong Chao, Ruo-Fei Tian, Wen-Jing Wang, Xin Fu, Peng Lin, Ting Guo, Bin Wang, Xiu-Xuan Sun, Xi Chen, Zhi-Nan Chen, Shi-Jie Wang and Hong-Yong Cui

Correspondence to: [znchen@fmmu.edu.cn](mailto:znchen@fmmu.edu.cn) (Z.N.C.), [kola_519@163.con](mailto:kola_519@163.con) (S.J.W.) and

[cui-hongyong@163.com](mailto:cui-hongyong@163.com) (H.Y.C.).

This file includes:

Supplementary Figures 1 to 9

Supplementary Tables 1 to 5


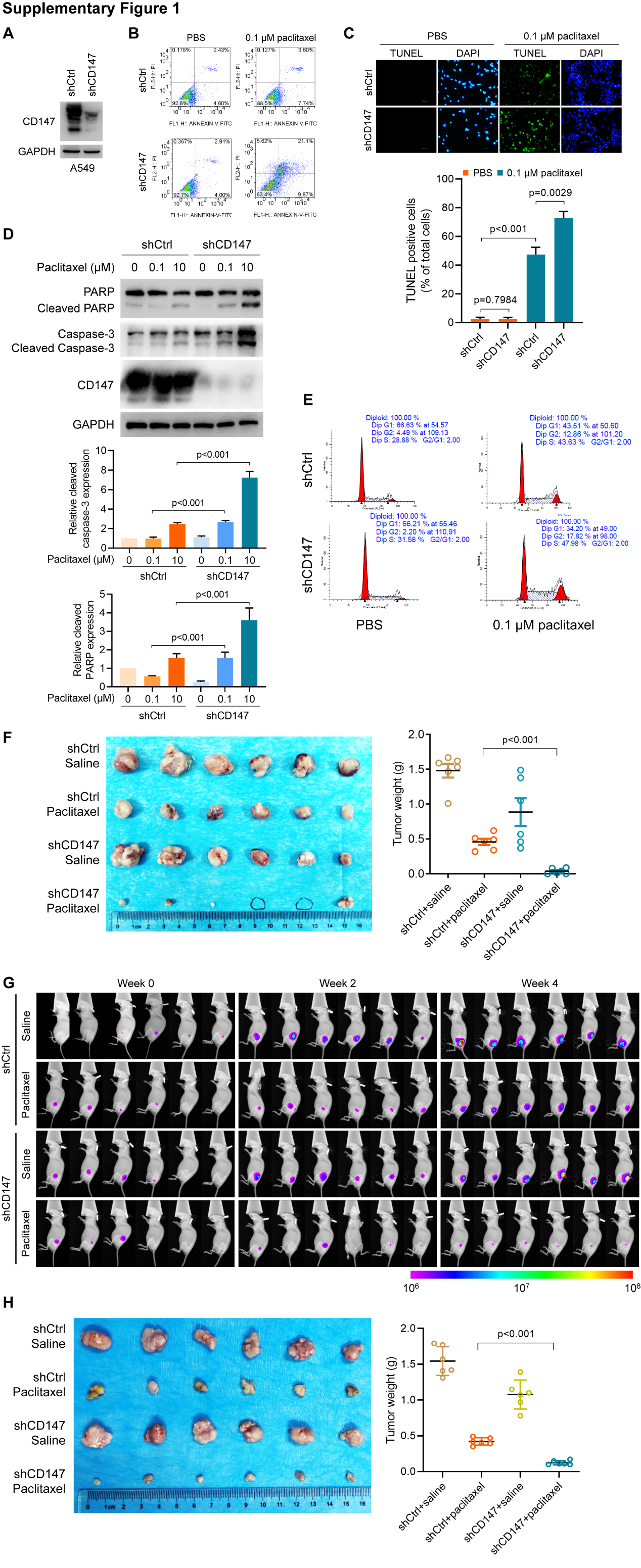


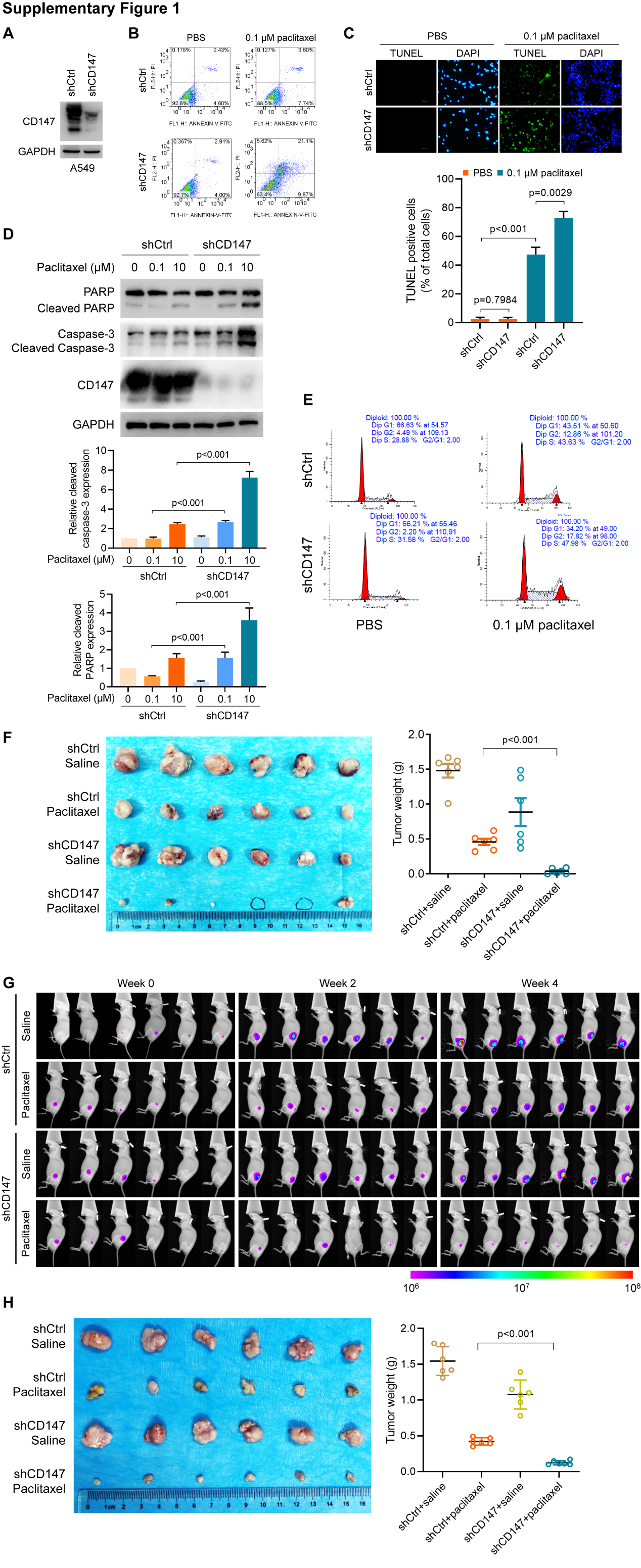


**Supplementary Figure 1: Silence of CD147 increases paclitaxel-induced cytotoxicity.**

**A** Western blot analysis of CD147 expression in A549 cells. **B** Apoptosis analysis of A549 cells by flow cytometry. **C** Representative images of TUNEL staining in A549 cells. Scale bar= 20 μm. The graph shows quantification of the percentage of TUNEL positive cells. **D** Western blot analysis of the indicated proteins in A549 cells. The graphs show semi-quantitative analysis of relative cleaved PARP and cleaved caspase-3 expression. **E** Cell cycle distribution of A549 cells treated with PBS or 0.1 μM paclitaxel. **F** Images of tumors formed after subcutaneous SK-OV-3 implantation. The graph shows quantification of tumor weight. **G** Nude mice with subcutaneous A549 xenografts were imaged in a Xenogen IVIS 200 system. **H** Images of tumors formed after subcutaneous A549 implantation. The graph shows quantification of tumor weight. The p-values in **C**-**D**, **F** and **H** were determined by using two-tailed Student’s t test.


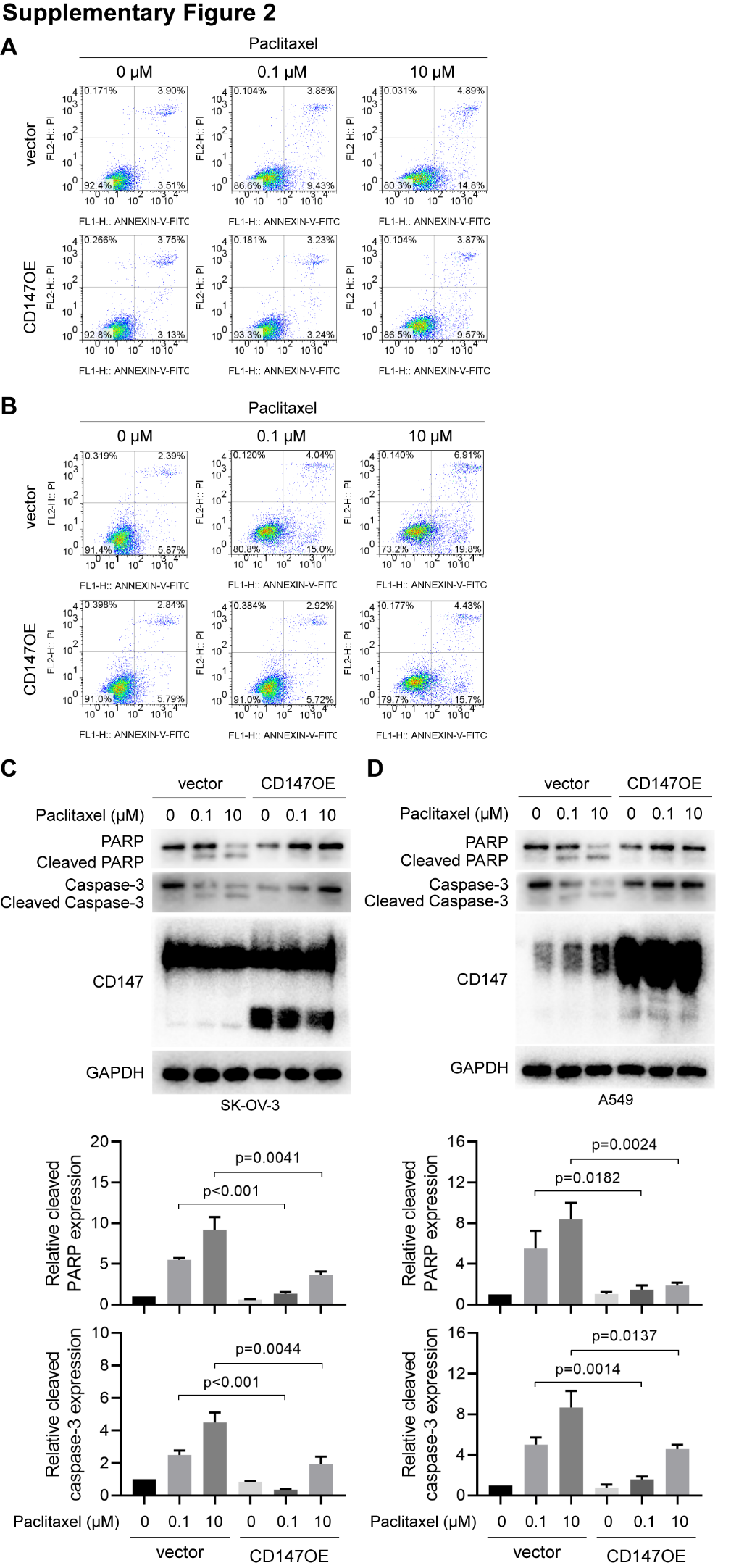


**Supplementary Figure 2: Overexpression of CD147 decreases sensitivity of cancer cells to paclitaxel.**

**A**-**B** Analysis of apoptosis by flow cytometry. SK-OV-3 (**A**) and A549 (**B**) cells were transfected with CD147-pcDNA3.1 or vector. **C**-**D** Western blot analysis of the indicated proteins in SK-OV-3 (**C**) and A549 (**D**) cells. The graphs show semi-quantitative analysis of relative cleaved PARP and cleaved caspase-3 expression. The p-values in **C**-**D** were determined by using two-tailed Student’s t test.


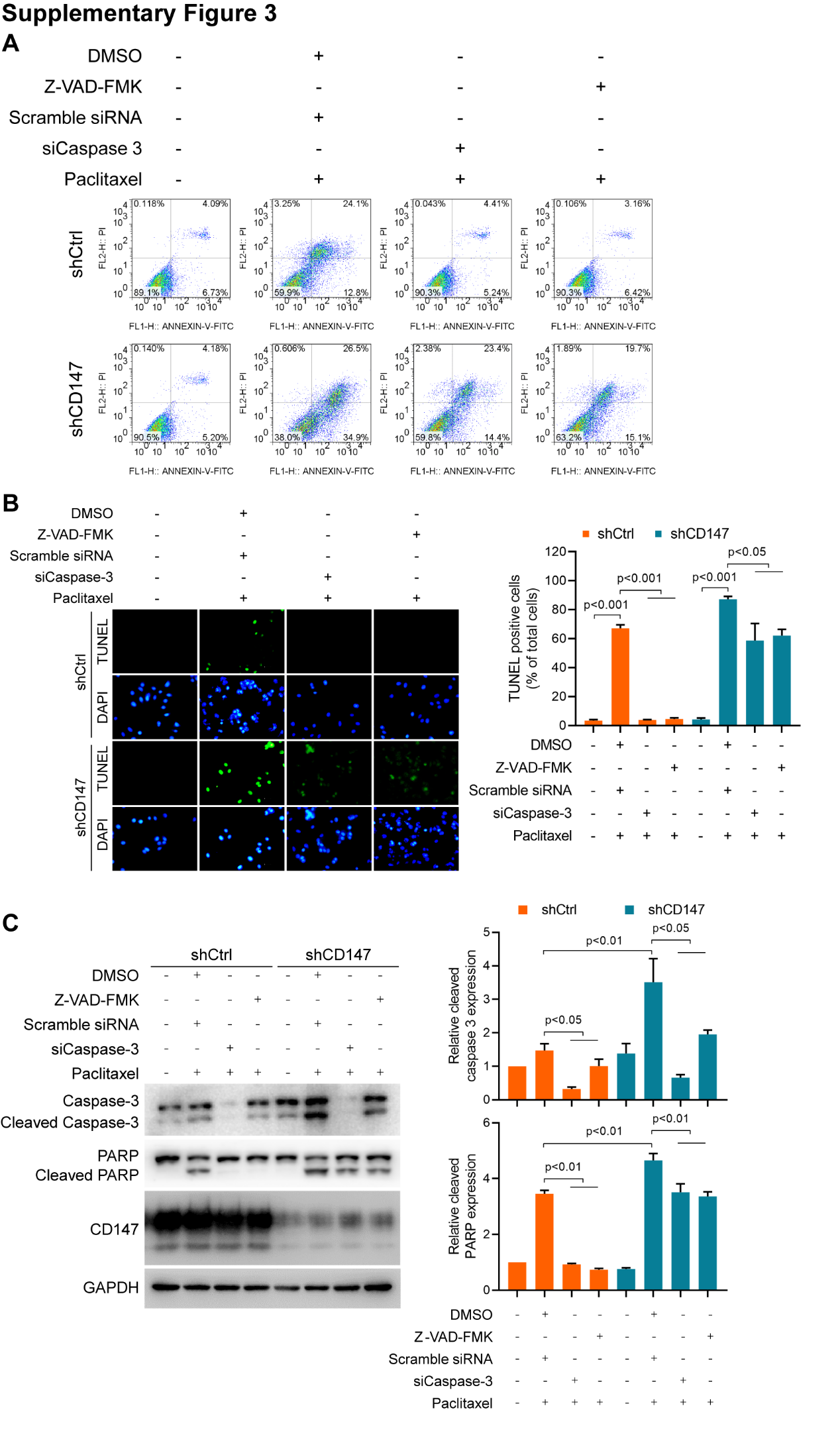


**Supplementary Figure 3: Caspase-3 mediates apoptosis induction by paclitaxel in CD147-silenced cells.**

**A** Analysis of apoptosis by flow cytometry. A549 cells with stably silencing of CD147 (shCD147) or control cells (shCtrl) were transfected with siRNAs targeting caspase 3 (siCaspase 3) or scramble siRNA alone or in combination with 40 μM Z-VAD-FAK treatment. **B** Representative images of TUNEL staining in A549 cells. Scale bar= 20 μm. The graph shows quantification of the percentage of TUNEL positive cells. **C** Western blot analysis of the indicated proteins in A549 cells. The graphs show semi-quantitative analysis of relative cleaved PARP and cleaved caspase-3 expression. The p-values in **B**-**C** were determined by using two-tailed Student’s t test.


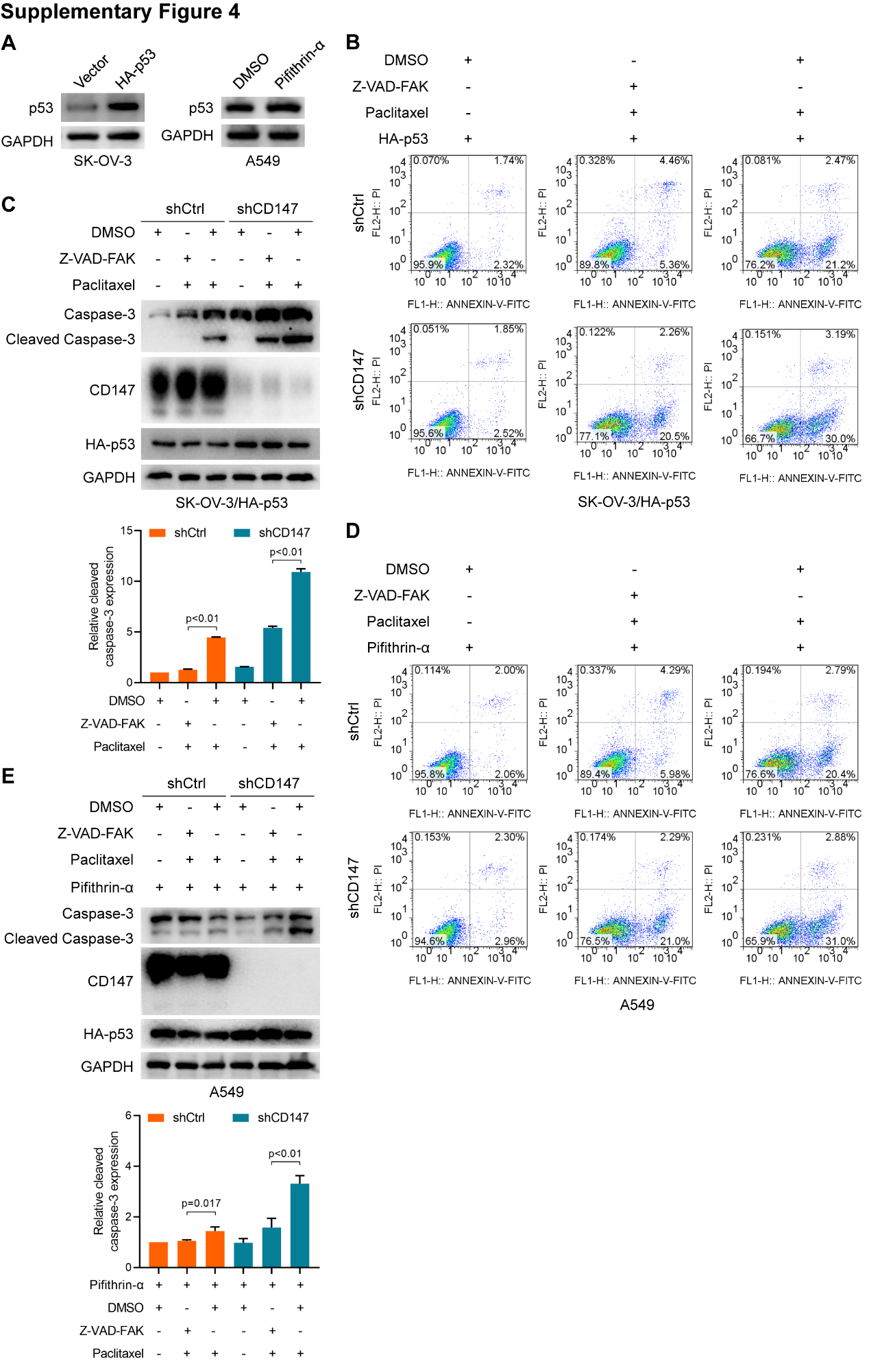


**Supplementary Figure 4: CD147 regulates paclitaxel response regardless of p53 status.**

**A** Western blot analysis of p53 expression in SK-OV-3 (left) and A549 (right) cells. **B** Apoptosis analysis of SK-OV-3/HA-p53 cells by flow cytometry. **C** Western blot analysis of the indicated proteins in SK-OV-3/HA-p53 cells. The graph shows semi-quantitative analysis of relative cleaved caspase-3 expression. **D** Apoptosis analysis of A549 cells by flow cytometry. **E** Western blot analysis of the indicated proteins in A549 cells. The graph shows semi-quantitative analysis of relative cleaved caspase-3 expression. The p-values in **C** and **E** were determined by using two-tailed Student’s t test.


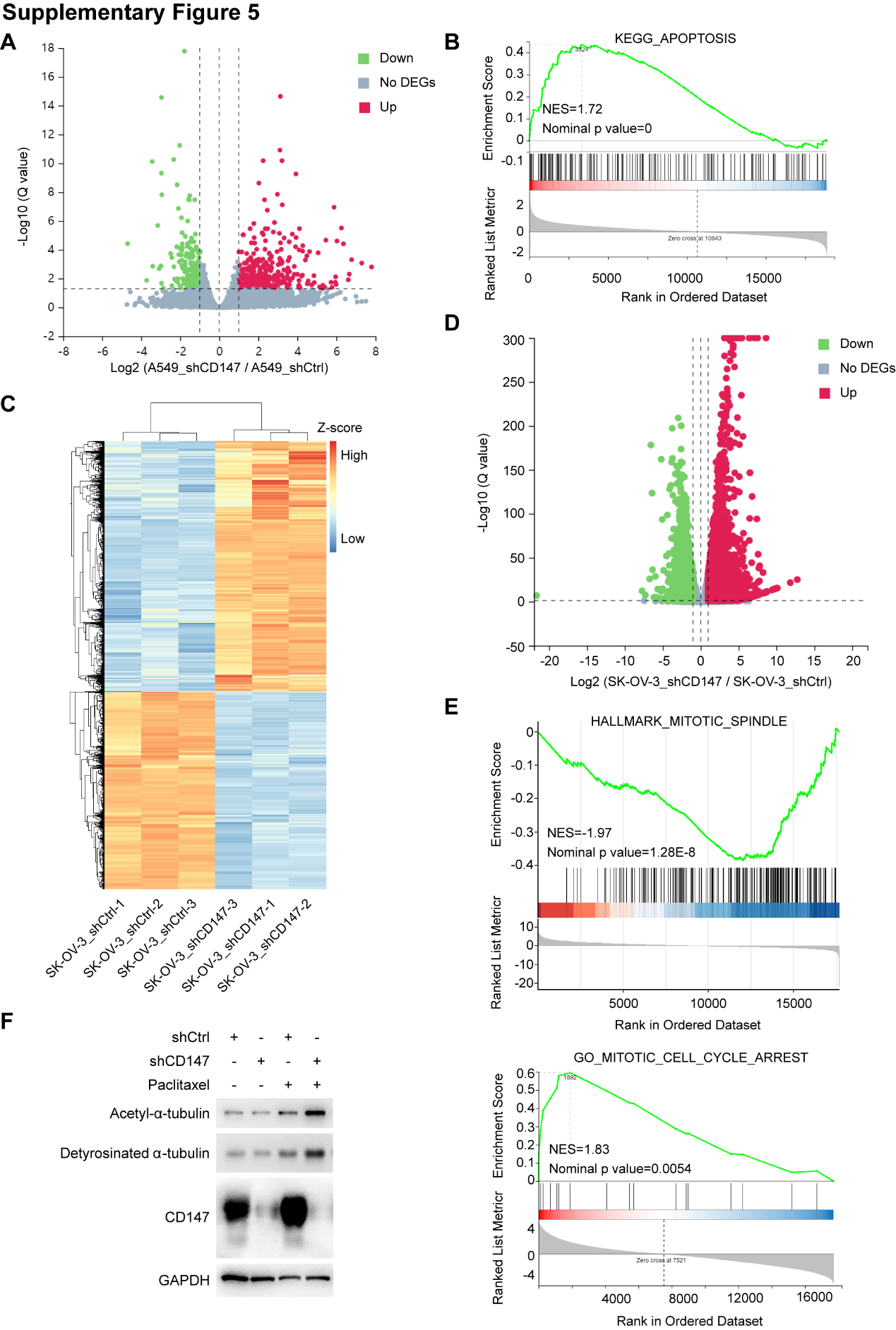


**Supplementary Figure 5: CD147 regulates paclitaxel-induced stabilization of microtubules.**

**A** A volcano plot displays DEGs in CD147 knockdown A549 cells (A549_shCD147) and control cells (A549_shCtrl). Cells were treated with 0.1 μM paclitaxel for 48 h. **B** Gene set enrichment analysis of DEGs in CD147 knockdown A549 cells (A549_shCD147) and control cells (A549_shCtrl). **C** Heatmap of DEGs in CD147 knockdown SK-OV-3 cells (SK-OV-3_shCD147) and control cells (SK-OV-3_shCtrl). Cells were treated with 0.1 μM paclitaxel for 48 h. **D** A volcano plot displays DEGs in SK-OV-3_shCD147 and SK-OV-3_shCtrl cells. **E** Gene set enrichment analysis of DEGs in SK-OV-3_shCD147 and SK-OV-3_shCtrl cells. **F** Western blot analysis of indicated proteins in CD147 knockdown cells and control cells treated with or without 0.1 μM paclitaxel.


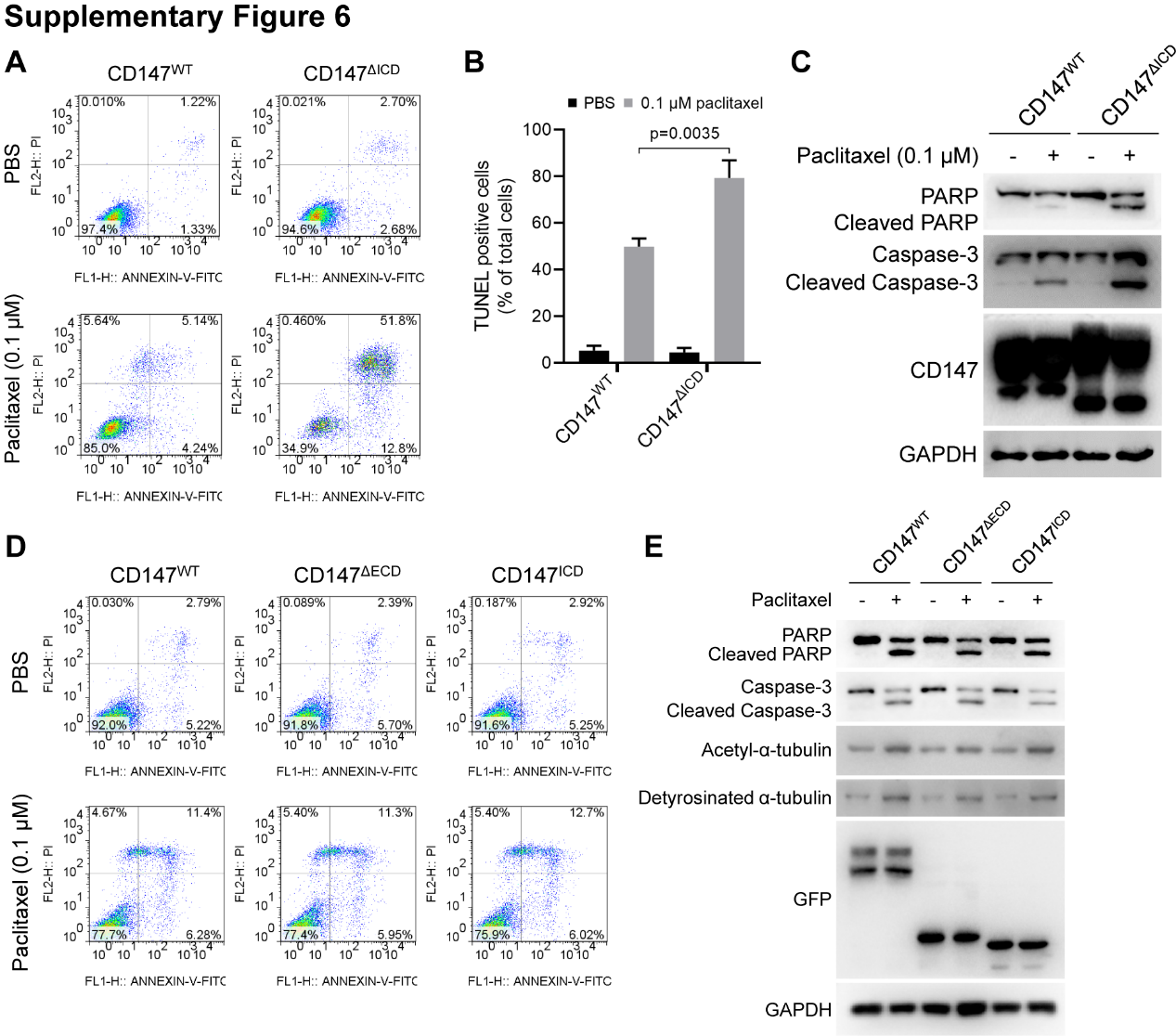


**Supplementary Figure 6: The intracellular domain of CD147 is responsible for CD147-regulated paclitaxel resistance.**

**A**-**C** CD147 knockdown A549 cells were transfected with CD147^WT^ or CD147^ΔICD^. Cells were treated with PBS or 0.1 μM paclitaxel. **A** Analysis of apoptosis by flow cytometry. **B** The graph shows quantification of the percentage of TUNEL positive cells. The p-value was determined by two-tailed Student’s t test. **C** Western blot analysis of indicated proteins. **D**-**E** CD147 knockdown A549 cells were transfected with CD147^WT^, CD147^ΔECD^ or CD147^ICD^. Cells were treated with PBS or 0.1 μM paclitaxel. **D** Analysis of apoptosis by flow cytometry. **E** Western blot analysis of indicated proteins.


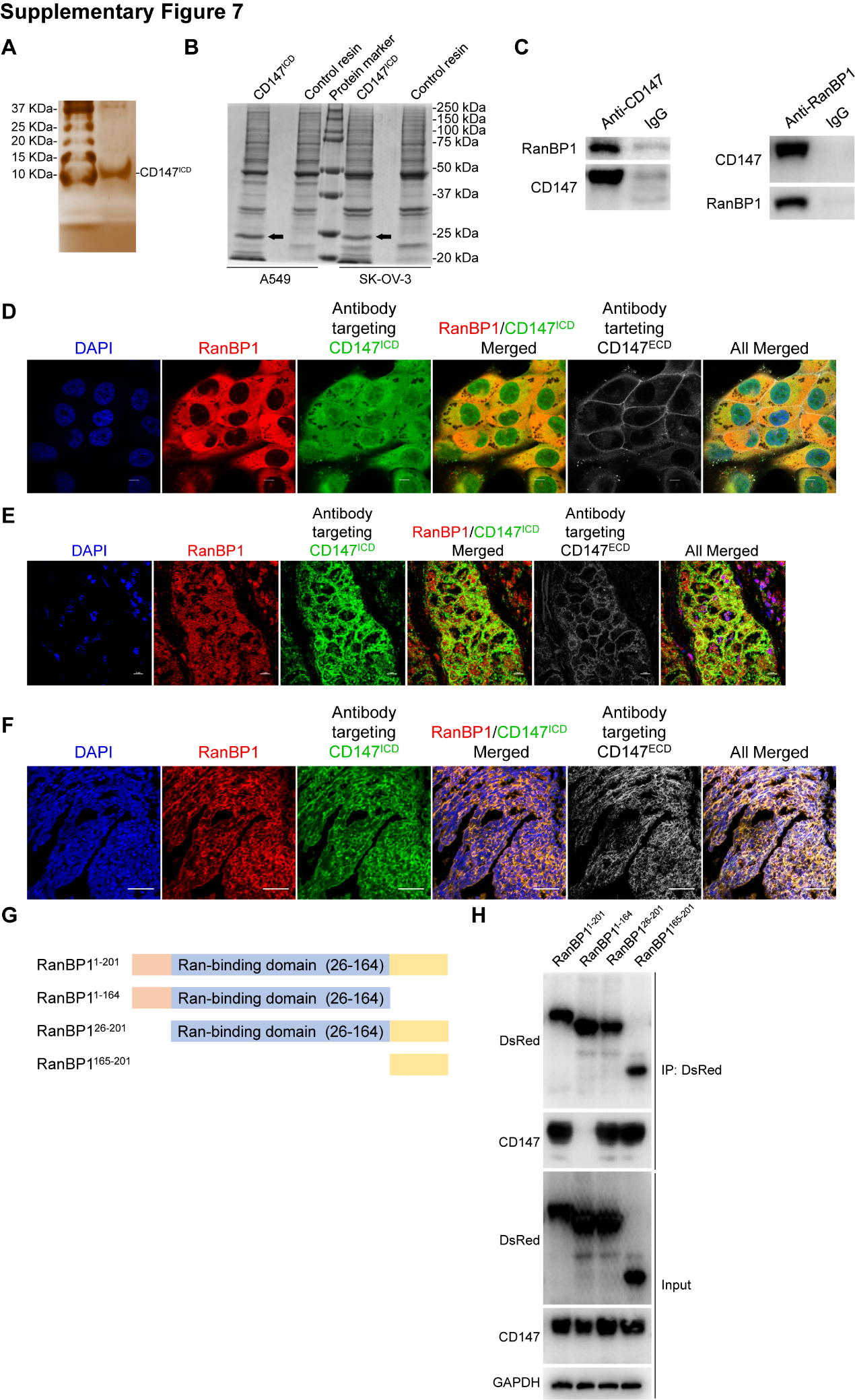


**Supplementary Figure 7: RanBP1 is an interacting partner for CD147.**

**A** Silver staining of purified CD147^ICD^. **B** Coomassie brilliant blue staining of the eluates from His pull-down assays. HisPur Cobalt Resin was used as a control to exclude nonspecific binding. The band corresponding to RanBP1 is indicated. **C** Western blot analyses of endogenous CD147 co-immunoprecipitated with endogenous RanBP1 in A549 cells. IgG was used as a control antibody for immunoprecipitation. **D** Representative images of immunofluorescent staining of endogenous CD147 and RanBP1 in SK-OV-3 cells. Scale bar=10 μm. **E** Representative images of immunofluorescent staining of endogenous CD147 and RanBP1 in NSCLC tissues. Scale bar=10 μm. **F** Representative images of immunofluorescent staining of endogenous CD147 and RanBP1 in ovarian cancer tissues. Scale bar=50 μm. **G** Schematic representation of the RanBP1 constructs. **H** Western blot analyses of various RanBP1 constructs co-immunoprecipitated with endogenous CD147 in A549 cells.


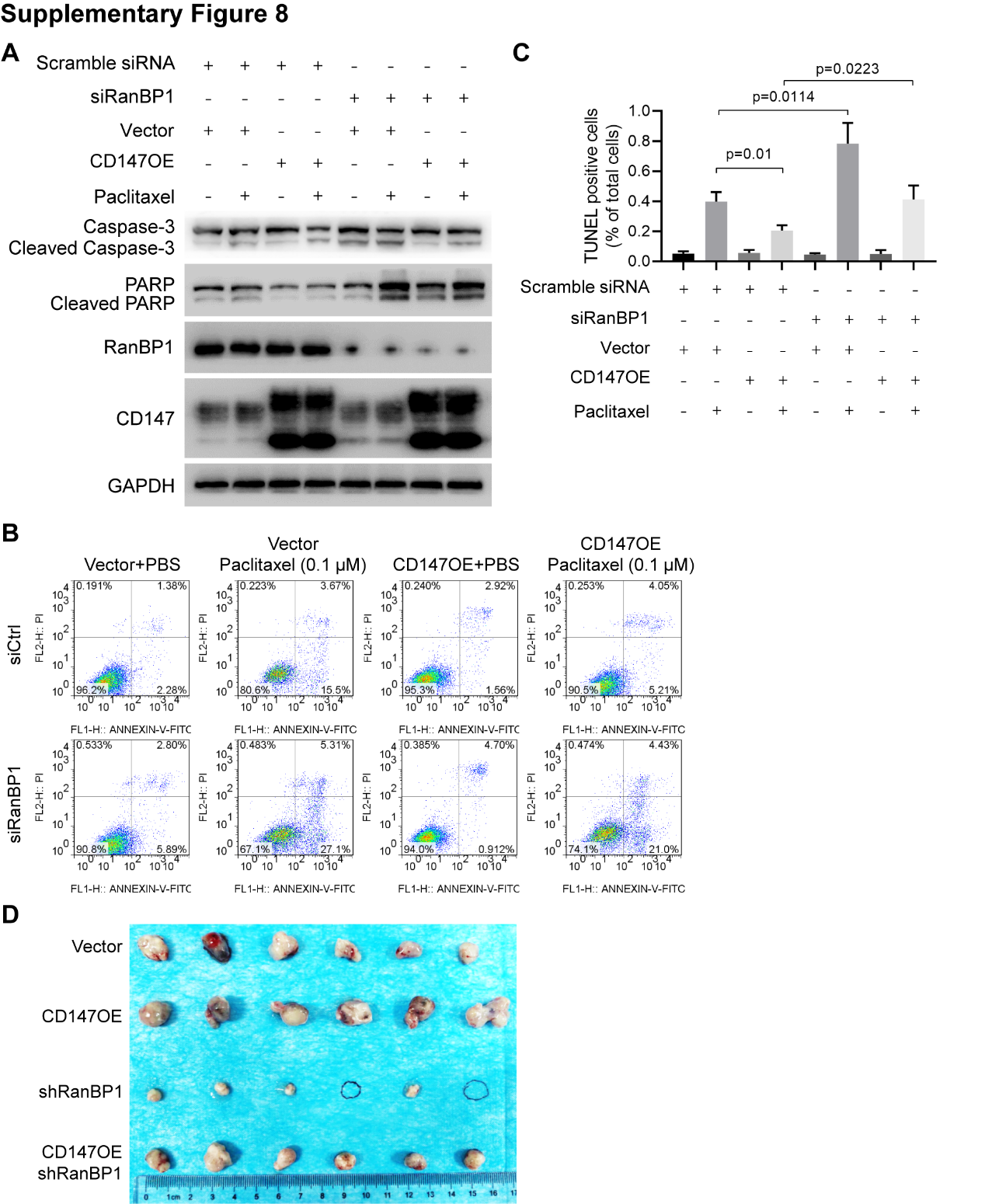


**Supplementary Figure 8: Overexpression of CD147 decreases paclitaxel-induced cytotoxicity via RanBP1.**

**A**-**C** A549 cells transfected with CD147-pcDNA3.1 or in combination with either siRNA targeting RanBP1 (siRanBP1) or scramble siRNA were treated with or without 0.1 μM paclitaxel. **A** Western blot analysis of the indicated proteins. **B** Analysis of apoptosis by flow cytometry. **C** The graph shows quantification of the percentage of TUNEL positive cells. The p-values were determined by using two-tailed Student’s t test. **D** Images of tumors formed after subcutaneous ovarian cancer implantation.


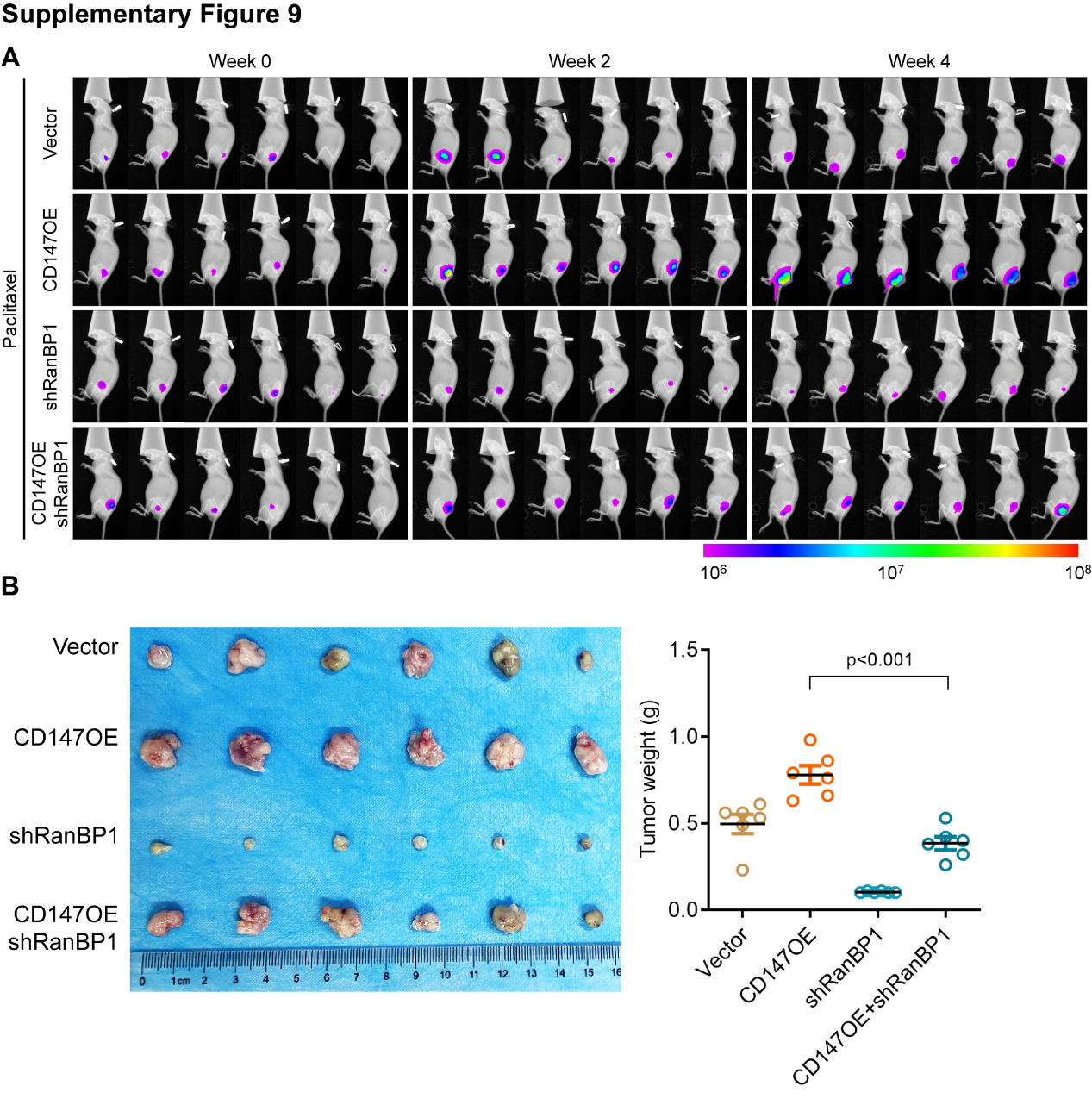


**Supplementary Figure 9: RanBP1 mediates the role of CD147 in paclitaxel response in subcutaneous A549 xenografts.**

**A** Nude mice with subcutaneous A549 xenografts were imaged in a Xenogen IVIS 200 system. **B** Images of tumors formed after subcutaneous A549 implantation. The graph shows quantification of tumor weight. The p-values were determined by two-tailed Student’s t test.

| **Supplementary Table 1 Distribution of selected characteristics in NSCLC cases** | | | |
| --- | --- | --- | --- |
| **Variables** | **Response, n=12** | **No response, n=13** | **P-value** |
| Age, mean y (SD) | 61.5 (13.1) | 60.7 (9.8) | 0.862^a)^ |
| Gender |  |  | 0.689^b)^ |
| Male, n (%) | 10 (83.3) | 10 (76.9) |  |
| Female, n (%) | 2 (16.7) | 3 (23.1) |  |
| Liver metastasis, n (%) | 3 (25.0) | 5 (38.5) | 0.471^b)^ |
| Smoking |  |  | 0.891^b)^ |
| Yes, n (%) | 8 (66.7) | 9 (69.2) |  |
| No, n (%) | 4 (33.3) | 4 (30.8) |  |
| CD147 expression |  |  | 0.048^b)^ |
| Negative, n (%) | 3 (25.0) | 0 (0) |  |
| Positive, n (%) | 9 (75.0) | 10 (76.9) |  |
| Strong positive, n (%) | 0 (0) | 3 (23.1) |  |

^a)^Two-tailed Student’s t-test.

^b)^Pearson's Chi-square test.

| **Supplementary Table 2 Distribution of selected characteristics in ovarian cancer cases** | | | |
| --- | --- | --- | --- |
| **Variables** | **Response, n=30** | **No response, n=20** | **P-value** |
| Age, mean y (SD) | 51.2 (9.4) | 50.1 (6.5) | 0.662^a)^ |
| Ascites, n (%) | 27 (90.0) | 15 (75.0) | 0.240^b)^ |
| Omentum metastasis, n (%) | 15 (50.0) | 17 (85.0) | 0.016^b)^ |
| Lymph node metastasis, n (%) | 17 (56.7) | 16 (80.0) | 0.129^b)^ |
| Differentiation degree |  |  | 0.722^b)^ |
| High, n (%) | 26 (86.7) | 18 (90.0) |  |
| Low, n (%) | 4 (13.3) | 2 (10.0) |  |
| CD147 expression |  |  | 0.035^b)^ |
| Negative, n (%) | 16 (53.3) | 4 (20.0) |  |
| Positive, n (%) | 7 (23.3) | 5 (25.0) |  |
| Strong positive, n (%) | 7 (23.3) | 11 (55.0) |  |

^a)^Two-tailed Student’s t-test.

^b)^Pearson's Chi-square test.

**Supplementary Table 3 Summary of LC/MS-MS analysis of CD147-RanBP1 interaction.**

| Sample ID | **CD147** | | | | | |  |  | **RanBP1** | | | | | |
| --- | --- | --- | --- | --- | --- | --- | --- | --- | --- | --- | --- | --- | --- | --- |
|  | CD147^ICD^-His_6_ | |  | | Resin | |  |  | CD147^ICD^-His_6_ | |  | | Resin | |
|  | # PSM | Area | | # PSM | | Area |  |  | # PSM | Area | | # PSM | | Area |
| A549-1 | 101 | 2.77E+08 | | 2 | | 2.26E+07 |  |  | 14 | 2.73E+09 | | 5 | | 2.17E+07 |
| A549-2 | 123 | 4.13E+09 | | N.D. | | N.D. |  |  | 13 | 3.48E+08 | | N.D. | | N.D. |
| A549-3 | 120 | 2.52E+09 | | N.D. | | N.D. |  |  | 10 | 4.06E+07 | | N.D. | | N.D. |
| SK-OV-3-1 | 56 | 3.46E+08 | | N.D. | | N.D. |  |  | 9 | 5.69E+07 | | N.D. | | N.D. |
| SK-OV-3-2 | 31 | 5.47E+08 | | N.D. | | N.D. |  |  | 7 | 6.36E+08 | | 1 | | N.D. |
| SK-OV-3-3 | 36 | 4.93E+08 | | N.D. | | N.D. |  |  | 6 | 8.12E+08 | | N.D. | | N.D. |

His pull-down was performed to identify CD147^ICD^-interacting proteins, where His_6_-tagged CD147^ICD^ protein was bait and the lysates from the A549 or SK-OV-3 cells were prey. The eluted proteins were identified by mass-spectrometry. # PSM, the number of peptide spectrum matches.

**Supplementary Table 4 List of RNAi sequences.**

| **RNAi ID** | **species** | **sense (5’ to 3’)** |
| --- | --- | --- |
| RanBP1-493 | Homo sapiens | GGACACUCAUGAGGACCAUTT |
| RanBP1-562 | Homo sapiens | GCCAAUAGUUUCUCUUCCUTT |
| RanBP1-762 | Homo sapiens | CCCUGAAGAUCUGUGCCAATT |
| Caspase-3-356 | Homo sapiens | CCCUGGACAACAGUUAUAATT |
| Caspase-3-855 | Homo sapiens | CACAGCACCUGGUUAUUAUTT |
| Caspase-3-941 | Homo sapiens | CCGACAAGCUUGAAUUUAUTT |
| CD147-720 | Homo sapiens | GUCGUCAGAACACAUCAACTT |
| CD147-858 | Homo sapiens | GUUCUUCGUGAGUUCCUCTT |
| CD147-1313 | Homo sapiens | CCCAUCAUACACUUCCUUCTT |

**Supplementary Table 5 Antibodies used in this study.**

| Antibodies | Source | Catalog number |
| --- | --- | --- |
| anti-CD147^ECD^ antibody | [1] | N/A |
| anti-CD147^ICD^ antibody | Santa cruz | Cat# sc-9754 |
| anti-GAPDH antibody | Sigma-Aldrich | Cat# HPA03648 |
| anti-PARP antibody | Cell Signaling Technology | Cat# 9542 |
| anti-Caspase-3 antibody | Cell Signaling Technology | Cat# 9662 |
| anti-Acetyl-α-tubulin antibody | Cell Signaling Technology | Cat# 5335 |
| anti-Detyrosinated-α-tubulin antibody | Merck | Cat# AB3201 |
| anti-Ran antibody | Proteintech | Cat# 10469-1-AP |
| anti-GFP antibody | Santa cruz | Cat# sc-9996 |
| anti-RanBP1 antibody | Abcam | Cat# ab97659 |
| Mouse IgG | Invitrogen | Cat# 31903 |
| Rabbit IgG | Invitrogen | Cat# 31235 |
| Goat anti-mouse IgG (H+L) antibody, Alexa Fluor 488 conjugate | Thermo Fisher Scientific | Cat# A-11001 |
| Goat anti-rabbit IgG (H+L) antibody, Alexa Fluor 488 conjugate | Thermo Fisher Scientific | Cat# A-11008 |
| Goat anti-Mouse IgG (H+L) antibody, Alexa Fluor Plus 555 conjugate | Thermo Fisher Scientific | Cat# A32727 |
| Goat anti-Rabbit IgG (H+L) antibody, Alexa Fluor Plus 555 conjugate | Thermo Fisher Scientific | Cat# A32732 |
| Donkey anti-Goat IgG (H+L) Highly Cross-Adsorbed Secondary Antibody, Alexa Fluor Plus 647 | Invitrogen | Cat# A32849 |

[1] Ku XM, Liao CG, Li Y, Yang XM, Yang B, Yao XY, Wang L, Kong LM, Zhao P, Chen ZN: Epitope mapping of series of monoclonal antibodies against the hepatocellular carcinoma-associated antigen HAb18G/CD147. *Scand J Immunol* 2007, 65:435-443.
